# Molecular features of interaction between VEGFA and anti-angiogenic drugs used in retinal diseases: a computational approach

**DOI:** 10.3389/fphar.2015.00248

**Published:** 2015-10-29

**Authors:** Chiara B. M. Platania, Luisa Di Paola, Gian M. Leggio, Giovanni L. Romano, Filippo Drago, Salvatore Salomone, Claudio Bucolo

**Affiliations:** ^1^Section of Pharmacology, Department of Biomedical and Biotechnological Sciences, School of Medicine, University of CataniaCatania, Italy; ^2^School of Engineering, University Campus BioMedicoRoma, Italy

**Keywords:** ranibizumab, bevacizumab, aflibercept, diabetic retinopathy, molecular dynamics

## Abstract

Anti-angiogenic agents are biological drugs used for treatment of retinal neovascular degenerative diseases. In this study, we aimed at *in silico* analysis of interaction of vascular endothelial growth factor A (VEGFA), the main mediator of angiogenesis, with binding domains of anti-angiogenic agents used for treatment of retinal diseases, such as ranibizumab, bevacizumab and aflibercept. The analysis of anti-VEGF/VEGFA complexes was carried out by means of protein-protein docking and molecular dynamics (MD) coupled to molecular mechanics-Poisson Boltzmann Surface Area (MM-PBSA) calculation. Molecular dynamics simulation was further analyzed by protein contact networks. Rough energetic evaluation with protein-protein docking scores revealed that aflibercept/VEGFA complex was characterized by electrostatic stabilization, whereas ranibizumab and bevacizumab complexes were stabilized by Van der Waals (VdW) energy term; these results were confirmed by MM-PBSA. Comparison of MM-PBSA predicted energy terms with experimental binding parameters reported in literature indicated that the high association rate (K_on_) of aflibercept to VEGFA was consistent with high stabilizing electrostatic energy. On the other hand, the relatively low experimental dissociation rate (K_off_) of ranibizumab may be attributed to lower conformational fluctuations of the ranibizumab/VEGFA complex, higher number of contacts and hydrogen bonds in comparison to bevacizumab and aflibercept. Thus, the anti-angiogenic agents have been found to be considerably different both in terms of molecular interactions and stabilizing energy. Characterization of such features can improve the design of novel biological drugs potentially useful in clinical practice.

## Introduction

**Figure 1:**
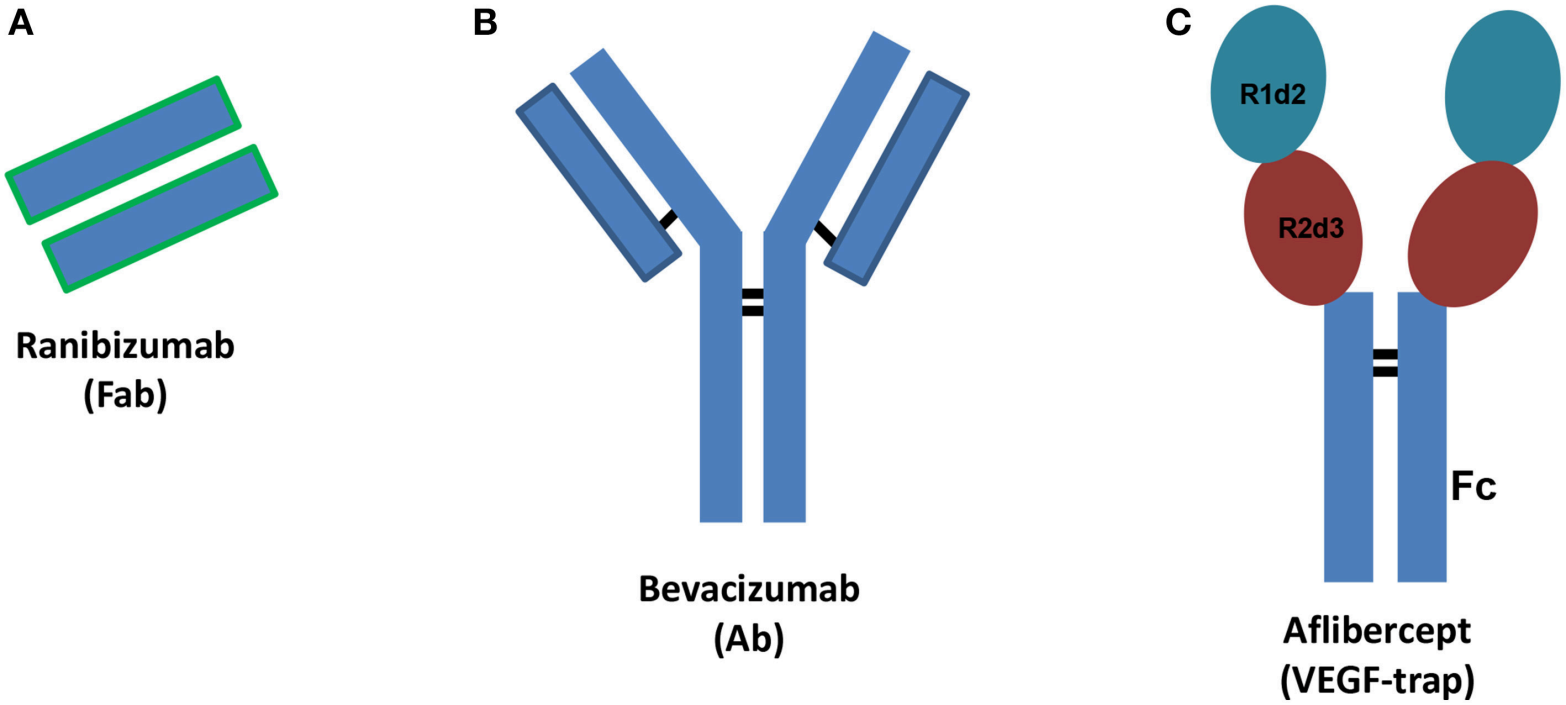
**Schematic structures of ranibizumab (A), bevacizumab (B), and aflibercept (C)**. Ab stands for antibody, Fab stands for fragment antigen binding, Fc stands for fragment crystallizable region. R1d2 stands for domain 2 of vascular endothelial growth factor receptor VEGFR1 and R2d3 stands for domain 3 of VEGFR2. Black bars correspond to inter-chain disulfide bridges.

Vascular endothelial growth factor (VEGF) plays a pivotal role in angiogenesis through activation of specific receptors (VEGFR). Among different VEGF isoforms, VEGFA (VEGF_165_) is the isoform that has principally been involved in angiogenesis. Angiogenesis is the physiological process of formation of new vessels from pre-existing ones and is crucial during development or tissue repair, but detrimental in some disease states. Therefore, in some pathological conditions angiogenesis inhibition is a relevant therapeutic goal (Carmeliet and Jain, 2011). Anti-VEGF agents are used to inhibit primary tumor and metastasis growth (Chung and Ferrara, 2011), both in adjuvant, and more recently, in neoadjuvant settings (Welti et al., 2013). Recently, anti-angiogenic agents have been used to treat ocular pathological conditions such as age-related macular degeneration (AMD) and diabetic macular edema (DME) (Holz et al., 2014; Stewart, 2014a). Diabetic retinopathy is a leading cause of vision loss of working-age adults, and DME is the most frequent cause of vision loss related to diabetes. AMD is a progressive multifactorial neurodegenerative disease that impairs the visual field. Currently, three VEGF inhibitors are used to treat retinal disorders characterized by neovessel formation: ranibizumab (Lucentis®), aflibercept (Eylea®) and bevacizumab (Avastin®), this latter is used off-label (Figure 1). Furthermore, several new anti-angiogenic agents are in clinical development (Teicher, 2011; Hanout et al., 2013; Li et al., 2014). Bevacizumab is a humanized monoclonal antibody, ranibizumab is the mutated Fab (Fragment antigen-binding) of the monoclonal antibody (Ab) originating bevacizumab and aflibercept is a fusion protein that works as decoy VEGF receptor (Figure 1). Ranibizumab has been developed for intravitreal injection and shows improved ocular pharmacokinetics (Xu et al., 2013) compared to bevacizumab. X-ray structures of VEGFA bound to ranibizumab (PDB: 1CZ8) (Chen et al., 1999) or Fab-bevacizumab (PDB: 1BJ1) (Muller et al., 1998) have been solved. The three dimensional structure of aflibercept is not available, albeit widely investigated. Aflibercept, also known as VEGF-trap, is a decoy receptor where two binding domains, the domain 2 (d2) of VEGFR1 and the domain 3 (d3) of VEGFR2 (from N-terminus to C-terminus of primary sequence) are connected to the fragment crystallizable region (Fc) of human immunoglobulin (Ig) (Holash et al., 2002). Throughout the text the binding domain of aflibercept is named “VEGFR1d2_R2d3.”

Iyer et al. (2010) reported the x-ray structure of VEGFR1 domain 2, as binary complex with VEGFB, while Leppänen et al. (2010) have solved the x-ray structure of VEGFR2 domain 2 and 3 (VEGFR2d2_d3) in complex with VEGFC, that we used for homology modeling of aflibercept's binding domain (VEGFR1d2_R2d3). However, *in silico* comparison of anti-VEGF/VEGFA complexes has not yet been carried out. Therefore, the aim of our study was to test the hypothesis that the three anti-VEGF/VEGFA complexes, involving ranibizumab, bevacizumab or aflibercept have different energy terms corresponding to different molecular and/or atomic interactions. To this end we carried out protein-protein docking with the software PyDock (Cheng et al., 2007; Jiménez-García et al., 2013), simulation of complexes with molecular dynamics (MD, GROMACS, Pronk et al., 2013) and molecular mechanics Poisson-Boltzmann surface area calculation (MM-PBSA) by using the g_mmpbsa tool (Kumari et al., 2014). Furthermore, we have added the analysis of the protein contact networks (Vishveshwara et al., 2009; Di Paola et al., 2013) on MD trajectories, to analyze the correlation of key topological properties over the time. Our results show that the three anti-angiogenic agents considerably differ, both in terms of molecular interactions and stabilizing energy; furthermore our *in silico* data are consistent with published experimental binding parameters (Papadopoulos et al., 2012).

## Methods

### Molecular modeling and protein-protein docking

Full protein sequences of Fab-bevacizumab binding domain and ranibizumab were retrieved from the DrugBank database (http://www.drugbank.ca, accession numbers: DB00112 and DB01270, respectively). The sequence of aflibercept binding domain (VEGFR1d2_R2d3) was built by connecting the sequences of domain 2 of human VEGFR1 and domain 3 of human VEGFR2:

SDTGRPFVEMYSEIPEIIHMTEGRELVIPCRVTSPNITVTLK KFPLDTLIPDGKRIIWDSRKGFIISNATYKEIGLLTCEATVNG HLYKTNYLTHRQTNTIIDVVLSPSHGIELSVGEKLVLNCTART ELNVGIDFNWEYPSSKHQHKKLVNRDLKTQSGSEMKKFLST LTIDGVTRSDQGLYTCAASSGLMTKKNSTFVRVHEDPIEGR.

Structures of Fab-bevacizumab and ranibizumab binding domains were modeled by means of the SwissModel web server (http://swissmodel.expasy.org/; Schwede et al., 2003) using, respectively, the x-ray structures of bevacizumab (PDB: 1BJ1) and ranibizumab (PDB: 1CZ8) in complex with the dimer of VEGFA as templates. This procedure included the modeling of short loops, which are lacking in the X-ray structures. The structure of VEGFR1d2_R2d3 was modeled using the x-ray structure of VEGFR2 in complex with VEGFC (PDB: 2X1W). Protein-Protein docking was carried out through the pyDockWEB (http://life.bsc.es/servlet/pydock/home/; Jiménez-García et al., 2013). The 3D coordinates of the ligand, the VEGFA dimer, were extracted from the crystal structure of VEGFA bound to ranibizumab (1CZ8). Homology models of Fab-bevacizumab and ranibizumab were set as PDB files of receptors. The complex VEGFR1d2_R2d3/VEGFA was built by using two randomly selected frames, belonging to relative conformational minimum of a preliminary MD of VEGFR1d2_R2d3 (Supplementary Material). Because of the low RMSD (0.06 nm), no differences were found between the two predicted complexes of the two analyzed frames. In order to evaluate the effectiveness of MD on VEGFR1d2_R2d3 structural optimization, the protein-protein docking of VEGFR1d2_R2d3 was also carried out using its starting model.

Given the 3D coordinates of two interacting proteins, pyDockWEB uses FTDock for generation of the predicted complexes by rigid-body docking (Gabb et al., 1997). No restrains have been applied in this stage. The predicted complexes were then evaluated by PyDock scoring function, which includes electrostatics, desolvation energy and limited Van der Waals (VdW) contributions (Cheng et al., 2007).

### Molecular dynamics simulation (MD)

**Figure 2:**
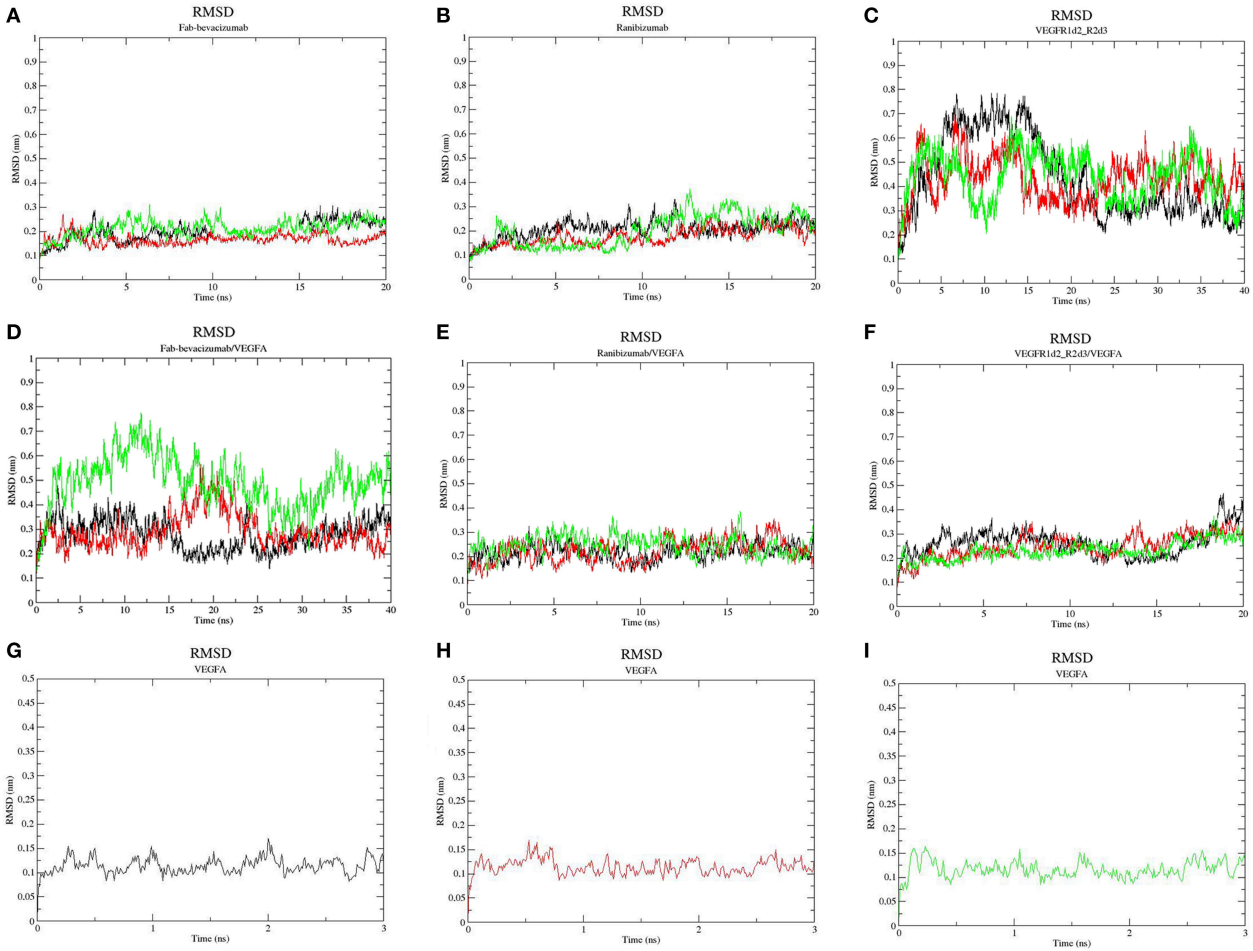
**Root-mean-square deviation (RMSD) profile of simulated molecules**. **(A)** ranibizumab; **(B)** Fab-Bevacizumab; **(C)** VEGFR1d2_R2d3; **(D)** ranibizumab/VEGFA complex; **(E)** Fab-bevacizumab/VEGFA complex; **(F)** VEGFR1d2_R2d3/VEGFA complex; **(G)** VEGFA replica 1; **(H)** VEGFA replica 2; **(I)** VEGFA replica 3. Black line corresponds to the first replica, red line corresponds to the second replica, green line corresponds to the third replica.

All-atom MD has been carried out with GROMACS 4.6 (Pronk et al., 2013), taking advantage of graphic processing unit (GPU) acceleration. Disulfide bridges in the object molecules were built as follows: VEGFA (Cys26-68Cys, Cys102-52Cys, Cys102-57Cys) in each monomer; Fab-bevacizumab and ranibizumab (Cys23-88Cys and Cys134-194Cys in L chain, Cys22-96Cys and Cys150-206Cys in H chain); VEGFR1d2_R2d3 (Cys30-79Cys in R1d2 and Cys189-125Cys in R2d3). Ionization state of residues was assigned at pH 7.4, OPLS-AA (Jorgensen et al., 1996) force field parameters were assigned to protein; thereafter, the following simulation protocol was applied: (i) building of the simulation system (explicit solvation with water molecules, SPC-E model (Berendsen et al., 1987), and neutralization with NaCl 150 mM); (ii) 2000 steps of steepest descent energy minimization (time-step = 1 fs); (iii) 500 ps of NVT equilibration (time-step = 1 fs); (iv) 500 ps of NPT equilibration (time-step = 1 fs); v. production runs (time-step = 2 fs). In order to use a 2 fs time-step we applied LINCS constraints (Hess et al., 1997) through the P-LINCS algorithm (Hess, 2008). Long-term electrostatic interactions were treated by applying the Particle Mesh Ewald (PME) method (Cheatham et al., 1995) (pme_order = 4; fourier spacing 0.16). Temperature was kept constant at 300 K with Berendsen thermostat applied to two groups (protein and non-protein), pressure was controlled by the Parinello-Rahman barostat (P = 1 bar; water compressibility 4.5 E-5 bar^−1^). Trajectory and energy outputs were collected every 10 ps. Three independent replicas for each MD were carried out: VEGFR1d2_R2d3 (40 ns), ranibizumab (20 ns), Fab-bevacizumab (20 ns), VEGFR1d2_R2d3/VEGFA complex (20 ns), ranibizumab/VEGFA complex (20 ns), Fab-bevacizumab/VEGFA complex (40 ns). VEGFA was simulated for just 3 ns, because the RMSD, secondary structure and cosine content indicated an equilibrated simulation. MD runs were launched on the EURORA machine (HPC-CINECA) using an average of 64 CPUs, 8 GPUs and 8 message passing interface (MPI) processes per run. Visual analysis of trajectories was carried out with Visual Molecular Dynamics VMD 1.9 (Humphrey et al., 1996); whereas analysis of MD simulations was carried out with the analysis package of GROMACS 4.6 (Pronk et al., 2013). We carried out Principal Component Analysis (PCA) of trajectories for characterization of principal collective motions of the analyzed molecules; such motions can identify the movement of a domain toward another domain of a given protein (Amadei et al., 1993). Each principal component is also named eigenvector to which an eigenvalue (~frequency) is associated. Eigenvectors were projected in the MD trajectories in order to visualize the collective motions of the protein, thereby reducing the dimensionality of trajectories. Because PCs with high cosine content are indicative of random diffusive movement (Hess, 2002; Neugebauer et al., 2002), in order to assess the correct conformational sampling of trajectories, cosine content of the first two PCs has been determined. A cosine content lower than 0.5 is considered as indicative of satisfactory conformational sampling. The number of contacts at protein-protein interface have been determined with g_mindist tool of GROMACS, residues were defined as interacting at 3.5 Å. High frequency H-bonds at the protein-protein interface have been analyzed using the HBonanza open source software (Feenstra et al., 1999). RMSD (Figures 2, **4**) calculations have been carried out by selecting Cα carbons of proteins and excluding the analysis of translational and rotational movements. The splitting of RMSD (**Figure 4**), of VEGFA/anti-VEGF trajectories, was obtained as follows: we launched the command g_rms using the gromacs index.ndx file in order to carry out the calculation on each component of complexes, the VEGFA and the anti-VEGFA, respectively; therefore RMSD profiles of complexes in Figure 2 are not the sum of split RMSD in **Figure 4**.

### Protein contact networks

The analysis was applied to 100 frames, 10 ps-spaced, taken from the last 10 ns of each MD replica; whereas MD of VEGFA was analyzed in the whole 3ns-replicas. Each frame was converted into an indirect, unweighted graph, whose nodes are the α-carbons and edges represent the mutual spatial distances between residues when their distance is within 4 and 8 Å, accounting to VdW interactions. This method, applied in a previous work to highlight the allosteric character of protein-ligand complexes (De Ruvo et al., 2012), accounts only for non-covalent bonds. After the definition of the protein contact network, the following global (whole structure) topological descriptors and a chemical-physical descriptor were derived:

1. *adeg* (average degree): the average number of contacts involving a single residue;
2. *asp* (average shortest path): the shortest path between two residues indicates the minimum number of steps (links) from one residue to another; *asp* is the average value over all residue pairs;
3. *E* (the Graph Energy): couples the graph global connectivity to the interactions in the represented molecular structure (Balakrishnan, 2004);
4. dG_solv_ (free solvation energy): quantitative descriptor of protein stability in water (Eisenberg and McLachlan, 1986), takes into account the overall energy gain of atoms passing from protein to water.

In order to assess whether MD reached a relative conformational minimum, the correlation pattern of topological descriptors and energy (dG_solv_) was analyzed over the time; the conformational minimum is characterized by a non-significant correlation coefficient of the variables. Furthermore, the protein contact network has been partitioned into clusters, according to a spectral clustering algorithm, which was previously applied to split the protein structure into functional modules. This method is able to detect functional domains in protein structures and complexes, along to the topological role of single residues that account for inter- and intra-module interaction (Tasdighian et al., 2014). Clustering results for each average structure of complexes and protein systems is represented as a partition color map of a two-dimensional matrix of cluster distribution along the sequence. Background (blue) corresponds to residues that do not belong to the same cluster. Residues belonging to the same cluster are represented with the same color. An interruption between cluster-sequence continuity, i.e., a residue shifting to a different cluster, which corresponds to a long-range contact, is represented as a projection termed “whisker.”

### MM-PBSA and energy decomposition

The MM-PBSA method calculates the three energetic terms of the binding free energy (Equation 1) (Kollman et al., 2000):

(1) $$\Delta {\text{G}}_{\text{binding}}=\;{\text{G}}_{\text{complex}}\;-\;({\text{G}}_{\text{protein}}+\;{\text{G}}_{\text{ligand}})$$

Free energy of either products or reagents is calculated taking in account three terms (Equation 2):

(2) $${\text{G}}_{\text{x}}\;=\;<\text{ E}{\;}_{\text{MM}}\;>\;+\;<\;{\text{G}}_{\text{solvation}}\;>\;-\text{TS}$$

Where E_MM_ is the vacuum potential energy and G_solvation_ is the free energy of solvation. E_MM_ includes E_bonded_ and E_non−bonded_ energies; E_non−bonded_ energy is the summation of Van der Waals (Lennard-Jones potential function) and electrostatic (Coulomb potential function) energy terms. G_solvation_, is characterized by the summation of two terms, G_polar_ and G_apolar_, which represent the electrostatic and the non-electrostatic term. G_polar_is calculated using a continuum implicit solvent model applying the Poisson-Boltzmann equation (Baker et al., 2001). The G_apolar_term results from the summation of G_cavity_ and G_VdW_ terms; G_cavity_ is the work done by the solute to create a cavity in the solvent, G_VdW_ is the attractive Van der Waals energy between solvent and solute. G_apolar_ accounts for the hydrophobic effect (Richmond, 1984). Single trajectory MM-PBSA calculations were carried out on each of the three MD replicas of complexes by using the g_mmpbsa tool (Kumari et al., 2014), which integrates functions from GROMACS and APBS (http://rashmikumari.github.io/g_mmpbsa/). The dielectric relative constant ε has been set to 3 for protein and 80 for water (Kukic et al., 2013). The solvent accessible surface area (SASA) method was used for calculation of G_apolar_; the surface tension constant γ was set to 0.022 KJ/mol Å^2^ (Nicholls et al., 1991). The current implementation of the MM-PBSA method in g_mmpbsa does not include calculation of the entropic term (S) in the equation 2; indeed, g_mmpbsa is unable to provide prediction of absolute binding free energy, providing mainly relative binding energies. For this reason, we use through the text the notation “ΔE_binding_” instead of “ΔG_binding_,” this latter would include entropy. The g_mmpbsa tool predicts the contribution of residues to the binding free energy by means of energy decomposition calculations; it allows the visualization of such results by means of common molecular visualization tools. The energy contribution is written in the b-factor field of the.pdb file and can be mapped in a 3D structure. MM-PBSA was applied to 100 frames, 10 ps-spaced, taken from the last 10 ns of each MD replica. The hardware used for these calculations was a Desktop PC (12 core Intel i7, 64 GB RAM, two GeForce GTX 680-SLI) launching 16 MPI processes per job with maximum available performance (8 h per calculation, 2% load memory).

### Statistical and graphical analysis

GraphPad (version 6; San Diego, CA, USA) was used to carry out statistical analysis and graph creation. Comparisons between two independent groups were made by unpaired Student's *t*-test; *p* < 0.05 were considered significant. RMSD graphs have been created with xmgrace (open GNU license). Figures have been created with OPEN PyMOL Molecular Graphics System, Schrödinger, LLC (New York, NY, USA).

## Results

### Homology modeling and protein-protein docking

Modeled structures of Fab-bevacizumab and ranibizumab showed low root mean square deviation (RMSD), respectively 0.003 nm and 0.002 nm, upon superimposition on PDB: 1BJ1 and PDB: 1CZ8 x-ray structures, respectively. The model of VEGFR1d2_R2d3 showed low RMSD (0.04 nm) upon superimposition on the corresponding template PDB:2X1W. VEGFR1d2_R2d3 was subjected to short all-atom MD simulation prior protein-protein docking with VEGFA (Supplementary Material, Figure S1). Protein-protein docking predictions were carried out with PyDock. The software was first validated by building complexes of Fab-bevacizumab and ranibizumab with VEGFA. RMSDs between the best scored Fab-bevacizumab/VEGFA and ranibizumab/VEGFA complexes and the correspondent x-ray structures were negligible (0.046 and 0.045 nm respectively). Subsequently we modeled the VEGFR1d2_R2d3/VEGFA complex. When compared to the starting model, MD simulation gave a better docking score for VEGFR1d2_R2d3 (Table 1). The complex VEGFR1d2_R2d3/VEGFA was compared to X-ray structures of VEGFR2 bound to VEGFC and VEGFA (PDB: 2X1W and PDB: 3V2A, respectively; Supplementary Material, Figure S2). Rough energetic evaluation of predicted complexes, obtained with PyDock, is shown in Table 1. Notice that VEGFR1d2_R2d3/VEGFA was stabilized by electrostatic interaction energy compared to Fab-bevacizumab/VEGFA and ranibizumab/VEGFA complexes, which were rather characterized by stabilizing desolvation and VdW energy terms.

**Table 1:** **Energetic contribution to PyDock score**.

**Complex**	**Electrostatic energy (kcal/mol)**	**Desolvation energy (kcal/mol)**	**VdW (kcal/mol)**	**RMSD (nm)**
Ranibizumab/VEGFA	−7	−24	−76	0.05 vs. 1CZ8
Fab-bevacizumab/VEGFA	−8	−20	−62	0.05 vs. 1BJ1
^*^VEGFR1d2_R2d3/VEGFA	−22	−9	19	0.30 vs. 2X1W 0.20 vs. 3V2A
^**^VEGFR1d2_R2d3/VEGFA	−11	−12	34	0.40 vs. 2X1W 0.50 vs. 3V2A

*VdW (Van der Waals interaction energy), RMSD (root-mean-square deviation)*.

* *Aflibercept binding domain optimized with MD*.

** *Aflibercept binding domain without structural optimization*.

### Molecular dynamics simulation (MD)

Three independent MD replicas of VEGFA, ranibizumab, Fab-bevacizumab, VEGFR1d2_R2d3 and of the corresponding 1:1 complexes with VEGFA were carried out. Ranibizumab and Fab-bavacizumab reached a relative minimum within 10 ns (Figures 2A,B), VEGFR1d2_R2d3 reached a relative minimum within 40 ns (Figure 2C). The great conformational fluctuation of unbound VEGFR1d2_R2d3 is mainly related to rotational freedom of the connecting hinge between domains R1d2 and R2d3, and to inter-conversion of turns to coil and vice-versa, of the loops in R1d2 domain (see also Supplementary Material, Figures S3–S5). Fab-bevacizumab/VEGFA complex was characterized in all three replicas by an RMSD higher than the other complexes (Figure 2E), despite each replica reached a relative minimum after about 10 ns simulation, as did the other complexes (Figure 2); VEGFA reached a relative conformational equilibrium in 3 ns (Figures 2G–I).

The secondary structures of unbound VEGR1d2_R2d3 and Fab-bevacizumab/VEGFA did not significantly change over the time (Supplementary Material, Figures S3–S8).

In order to characterize the principal collective motions of proteins and their respective macromolecular complexes we carried out PCA of covariance matrix of trajectories. The first six eigenvectors explained about 99% of variance of each simulation. The first principal component (PC1) was projected into the MD trajectory of each simulated molecule (videos in Supplementary Material). In order to verify the correct conformational sampling of MD cosine content of the first two eigenvectors of each trajectory was analyzed (Table 2). Because cosine content of PC1 and PC2 was lower than 0.4 (cut-off 0.5), we assumed that conformational sampling of all MD runs was satisfactory.

**Table 2:** **Cosine content of the first two PCs of molecular dynamics simulations**.

	**Replica I**		**Replica II**		**Replica III**	
	**PC1**	**PC2**	**PC1**	**PC2**	**PC1**	**PC2**
VEGFA	0.35	0.01	0.22	0.05	0.36	0.02
Ranibizumab	0.36	0.15	0.02	0.25	0.04	0.32
Fab-bevacizumab	0.35	0.24	0.50	0.20	0.36	0.02
VEGFR1d2_R2d3	0.18	0.14	0.01	0.02	0.46	0.01
Ranibizumab/VEGFA	0.01	0.32	0.13	0.30	0.35	0.15
Fab-bevacizumab/VEGFA	0.34	0.03	0.42	0.16	0.37	0.13
VEGFR1d2_R2d3/VEGFA	0.10	0.36	0.36	0.38	0.24	0.32

*PC stands for Principal Component*.

### Protein contact networks analysis

To confirm that the systems were analyzed at relative conformational minimum, topological descriptors of protein contact network were also analyzed. The results of correlation analysis between topological descriptors and time for complexes are reported in Table 3 (for unbound systems see also Supplementary Material, Table S1). The energetic descriptor dG_solv_ did not change over the time, such that the correlation dG_solv_ vs. time was not significant.

**Table 3:** **Correlation analysis of topological parameters of anti-vegf/VEGFA complexes**.

	**Ranibizumab/VEGFA**					**Fab-bevacizumab/VEGFA**					**VEGFR1d2_R2d3/VEGFA**				
	**t**	**adeg**	**asp**	**E**	**dG_*solv*_**	**t**	**adeg**	**asp**	**E**	**dG_solv_**	**t**	**adeg**	**asp**	**E**	**dG_solv_**
t	–	0.20	0.61	0.07	−0.12	–	−0.12	0.29	−0.20	−0.30	–	−0.60	0.35	−0.54	0.46
adeg	–	–	−0.18	0.94	−0.47	–	–	−0.13	0.91	−0.33	–	–	−0.63	0.93	−0.51
asp	–	–	–	−0.28	0.07	–	–	–	−0.17	0.05	–	–	–	−0.61	0.34
E	–	–	–	–	−0.50	–	–	–	–	−0.28	–	–	–	–	−0.45
dG_solv_	–	–	–	–	–	–	–	–	–	–	–	–	–	–	–

In contrast, some topological descriptors showed correlations with time and/or each other. Indeed, the average shortest path *asp* positively correlated with time (0.61), while the average degree *adeg* and the graph energy *E* showed, respectively, a positive and a negative correlation in ranibizumab complex. The topological descriptors *adeg* and *asp* showed negative correlations (-0.60 and -0.54, respectively) with time in VEGFR1d2_R2d3. Finally, strong correlation between *E* and *adeg* for all complexes indicated that the graph energy *E* describes the overall connectivity energy.

**Figure 3:**
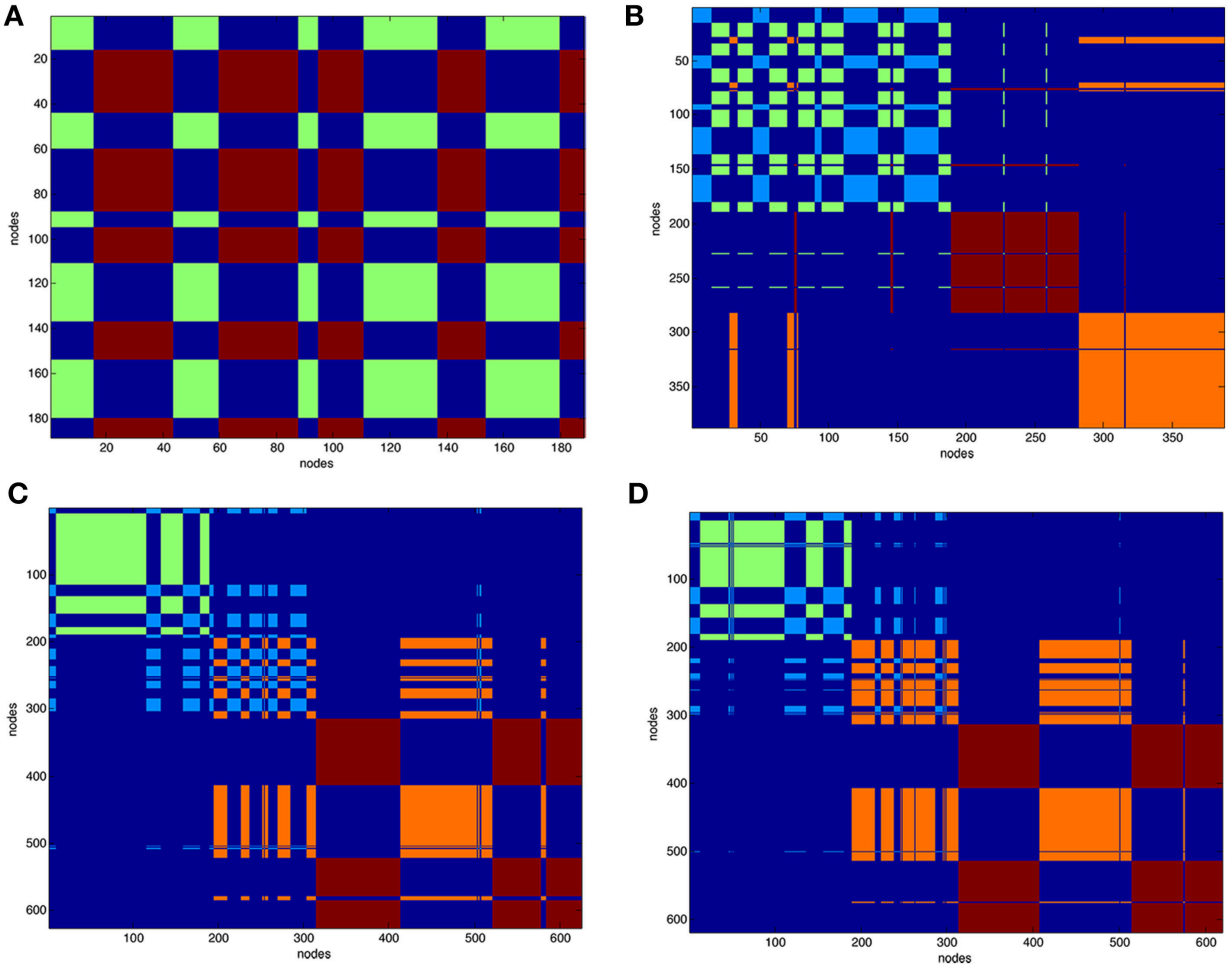
**Clustering of protein contact networks**. Distribution of clusters along sequence in a matricial space. Partition color maps of **(A)**. VEGFA (200 nodes), **(B)**. VEGFR1d2_R2d3/VEGFA, **(C)**. Fab-bevacizumab/VEGFA, **(D)**. ranibizumab/VEGFA. The first 200 nodes in complexes corresponds to bound VEGFA. In **(A)** green and light blue clusters corresponds to VEGFA. In **(B–D)** dark red and orange are clusters of anti-VEGF. Residues (nodes) belonging to the same cluster have the same color, long projections “whiskers” in the map represent residues shifting to a different cluster with respect to that of neighbors in sequence. The background is dark blue and characterizes residues that are not in the same cluster.

After partitioning of the protein contact network into clusters the structure of the complexes was represented as functional modules. Figure 3 reports the clustering of VEGFA (partitioned in two clusters) and all complexes (partitioned in four clusters). Clustering of unbound anti-VEGFs is reported in Supplemental Material (Figure S9).

The VEGFA partition in two clusters revealed that they were intermingled (Figure 3A). VEGFR1d2_R2d3/VEGFA partition in four clusters (Figure 3B) revealed a conserved network for VEGFA and two distinct domains corresponding to R1d2 and R2d3, with a lot of long-range interactions with VEGFA (Figure 3B). The partition in four clusters of Fab-bevacizumab/VEGFA (Figure 3C) and ranibizumab/VEGFA (Figure 3D), revealed for bound VEGFA a partition that differ from that of unbound VEGFA, while some whiskers projected from VEGFA to Fab-bevacizumab and ranibizumab modules; furthermore, a greater number of long-range interactions was formed between ranibizumab and VEGFA in comparison to Fab-bevacizumab.

### MM-PBSA calculation

An in-depth analysis of MD data was carried out with MM-PBSA calculations. GROMACS output files were directly analyzed with the g_mmpbsa tool. The estimated binding energy ΔE_binding_ and its contributing terms were compared to experimental binding and kinetic parameters (Table 4; Papadopoulos et al., 2012).

**Table 4:** **MM-PBSA results compared to experimental binding parameters**.

**Complex**	**Binding parameters**			**MM-PBSA energy terms (KJ/mol)**				
	**K_on_/10^5^ (M^−1^ s^−1^)**	**K_off_/10^−5^ (s^−1^)**	**K_D_ (pM)**	**ΔE_binding_**	**ΔE_VdW_**	**ΔE_electrostatic_**	**ΔG_Polar_**	**ΔG_Apolar_**
Ranibizumab/VEGFA	1.60	0.73	46	−7.0±40	−4.8±5	−1.0±20	410 ± 30	−5.2±7
Fab-bevacizumab/VEGFA	5.30	3.10	58	−8.7±30	−3.2±10	−2.2±20	343 ± 30	−5.6±7
VEGFR1d2_R2d3/VEGFA	410	2.01	0.49	−14.0±90	−3.7±50	−14.3±1.0	1050 ± 100	−7.0±40

*Kinetic and binding parameters are from Papadopoulos et al. (2012)*.

The relationship between K_*D*_ and binding energy is given by: ΔG = RT ln K_D_ at 1M concentration, where R is the ideal gas constant and T is the absolute temperature. In Table 4 ΔE_binding_ is reported instead of ΔG_binding_, because the entropic term is not included. The comparison of experimental K_*D*_ with predicted ΔE_binding_ confirmed a most favorable binding energy for VEGFR1d2_R2d3 compared to ranibizumab and Fab-bevacizumab bound to VEGFA. The differences in ΔE_binding_ for ranibizumab/VEGFA and Fab-bevacizumab/VEGFA vs. VEGFR1d2_R2d3/VEGFA were significant (*t*-test respectively *p* = 0.003 and *p* = 0.004). Because ΔE_binding_ results as summation of different energy terms, we analyzed each single energy term; interestingly, there was a correlation between experimental K_D_ and ΔG_Apolar_ (apolar contribution to desolvation energy), suggesting that the hydrophobic effect substantially accounts for affinity. As reported in Table 1, a relevant electrostatic stabilization was predicted by PyDock for the VEGFR1d2_R2d3/VEGFA complex, a data confirmed by MM-PBSA (Table 4). Furthermore, the higher K_on_ of aflibercept (410 M^−1^ s^−1^), as compared to ranibizumab (1.6 M^−1^ s^−1^) and bevacizumab (5.6 M^−1^ s^−1^), was consistent with the favorable electrostatic component of the binding energy, because the association rate of proteins is known to be related to electrostatic forces (Wade et al., 1998; Zhou, 2001; Pan et al., 2013). Polar contribution to the solvation energy (and to the whole ΔE_binding_) is positive, i.e., unfavorable, because of the polar/charged residue transition to a more hydrophobic environment; however, despite the ΔG_polar_ is more positive for VEGFR1d2_R2d3/VEGFA than for ranibizumab/VEGFA and Fab-bevacizumab/VEGFA, ΔE_electrostatic_-ΔG_polar_ gives a favorable gain only forVEGFR1d2_R2d3/VEGFA. Compensation of favorable electrostatic and unfavorable polar desolvation energy terms is commonly found in complex formation, while most stabilization arises from non-polar interactions and hydrophobic effect (Ozboyaci et al., 2011; Spiliotopoulos et al., 2012; Kar et al., 2013). Another information provided by the PyDock prediction was the substantial favorable VdW energy term for ranibizumab/VEGFA and Fab-bevacizumab/VEGFA (Table 1); this observation was confirmed by MM-PBSA (Table 4). Furthermore, VdW energy did not appear to be correlated to any kinetic binding parameter.

### Dissociation rate of complexes

**Figure 4:**
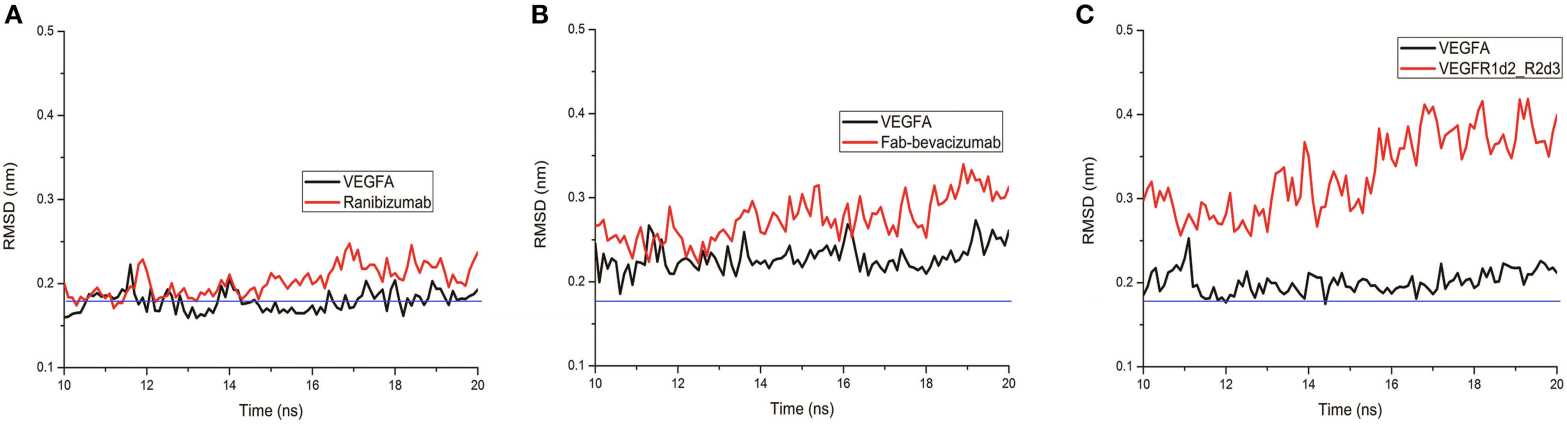
**Representative graphs of split RMSD of VEGFA (black line) and anti-VEGF (red line) binding domains. (A)** Split RMSD for the ranibizumab/VEGFA complex; **(B)** split RMSD for Fab-bevacizumab/VEGFA complex; **(C)** split RMSD for VEGFR1d2_R2d3/VEGFA complex.

**Figure 5:**
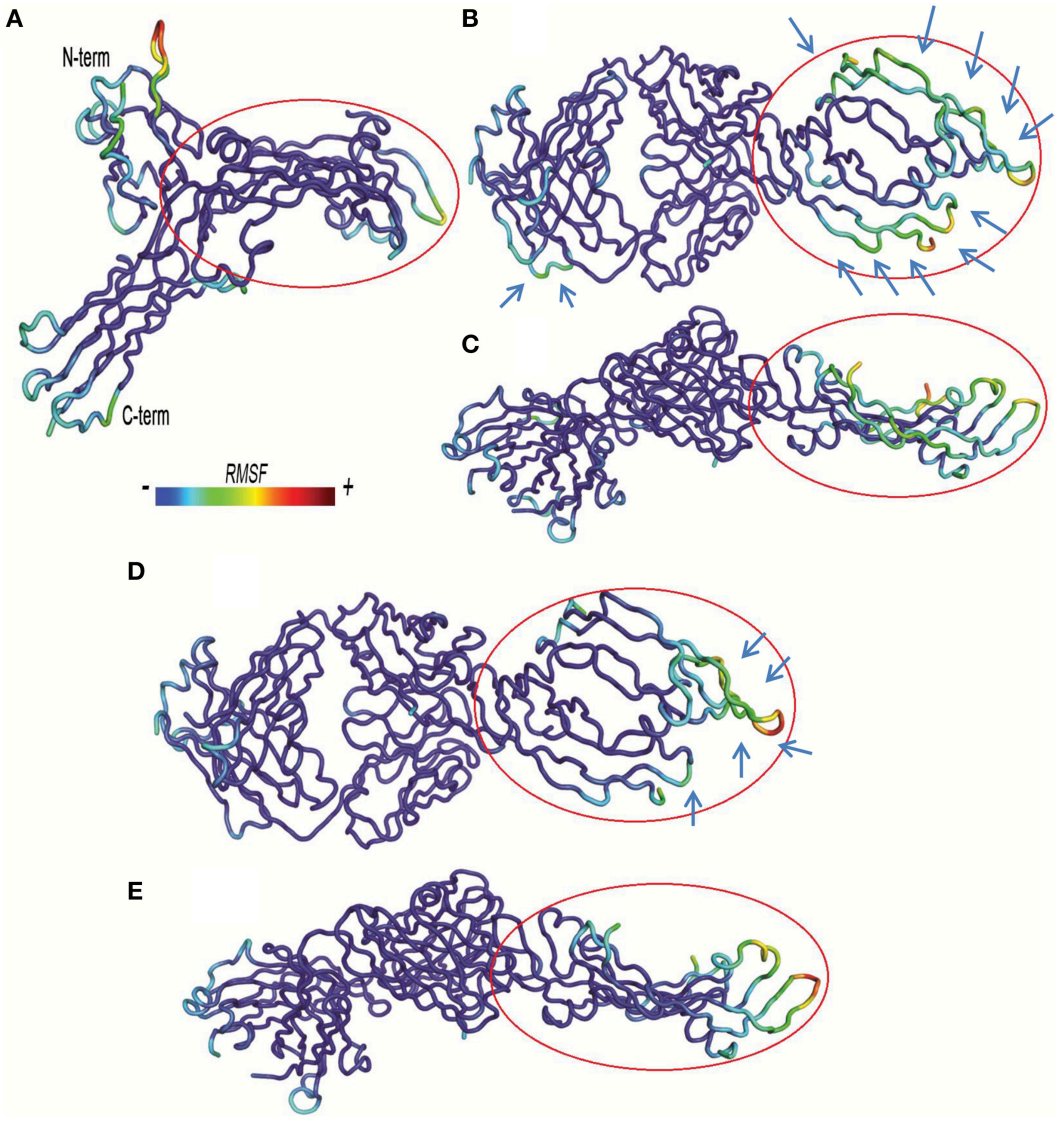
**Residue based-root mean square fluctuations (RMSF) of complexes**. RMSF increases from blue to red color. Bound VEGFA molecule is highlighted with a red circle. **(A)** VEGFR1d2_R2d3/VEGFA. **(B,C)** Fab-bevacizumab/VEGFA, respectively from top and side view. **(D,E)** Ranibizumab/VEGFA respectively, from top and side view.

A lower dissociation rate is reported for ranibizumab/VEGFA compared to the other two complexes (Papadopoulos et al., 2012). The number of contacts at protein-protein interface of complexes at 3.5 Å were determined by the g_mindist tool of GROMACS, while the number of H-bond was assessed by Hbonanza. The number of contacts resulted as follows: Ranibizumab/VEGFA, 480.7 ± 0.5; Fab-bevacizumab/VEGFA, 436.5 ± 0.4; VEGFR1d2_R2d3/VEGFA, 289.9 ± 1.8. The number of H-bonds, whose location is reported in Table 5, were: Ranibizumab/VEGFA, 10; Fab-bevacizumab/VEGFA, 5; VEGFR1d2_R2d3/VEGFA, 4. This analysis suggested that the complex Ranibizumab/VEGFA might be more stable than the other two complexes, in terms of residency time. To test this hypothesis we further analyzed the profiles of complexes by splitting the RMSD for each interacting protein. As shown in Figure 4, ranibizumab in the complex ranibizumab/VEGFA had the lowest RMSD, Fab-bevacizumab in the complex Fab-bevacizumab/VEGFA had an intermediate RMSD, VEGFR1d2_R2d3 in the complex VEGFR1d2_R2d3/VEGFA had the highest RMSD. Further analysis was carried out by calculating the residue-based root mean square fluctuation (RMSF) over the whole simulations. The residue-based RMSF of complexes was visualized into 3D structures (Figure 5).

**Table 5:** **High frequency H-bonds at anti-VEGF/VEGFA interfaces**.

	**Anti-VEGF/VEGFA**
Ranibizumab	Tyr102/Glu93; Tyr101/Glu93; Tyr101/Glu79; Ser106/His90; Tyr99/Glu87; Tyr96/Glu87; Tyr34/Glu89; Thr53/Glu89; Tyr54/Tyr21; Trp50/His89.
Fab-bevacizumab	His101/Glu93; Tyr102/Glu93; Ser106/His90; Thr53/Gln89; Tyr54/Tyr21.
VEGFR1d2_R2d3	Arg96/Asp93; Gln97/Lys107; Glu73/Arg105; Glu73/Arg103.

*In the couple X/Y, X, and Y are respectively the residues of anti-angiogenic agent and VEGFA, involved in the H-bond*.

The visualization of RMSF confirmed less structural fluctuation of ranibizumab/VEGFA compared to Fab-bevacizumab/VEGFA, consistent with their difference in experimental K_off_, and RMSD profiles. The representation of residue-based RMSF of VEGFR1d2_R2d3/VEGFA showed that VEGFA is mainly stabilized at the contact surface with domain 2 and domain 3 (Figure 5A); a high degree of fluctuation, however, detectable out of the contact region, may account for the conformational flexibility of VEGFR1d2_R2d3. Ranibizumab/VEGFA showed less conformational flexibility compared to both VEGFR1d2_R2d3/VEGFA and Fab-bevacizumab/VEGFA, suggesting a higher conformational stability of the complex (Zheng et al., 2006). The lower K_off_ of ranibizumab/VEGFA is in accordance with the dependency of the residence time τ (1/K_off_) on the conformational stabilization of the complex (Copeland, 2011).

### Energy decomposition

**Figure 6:**
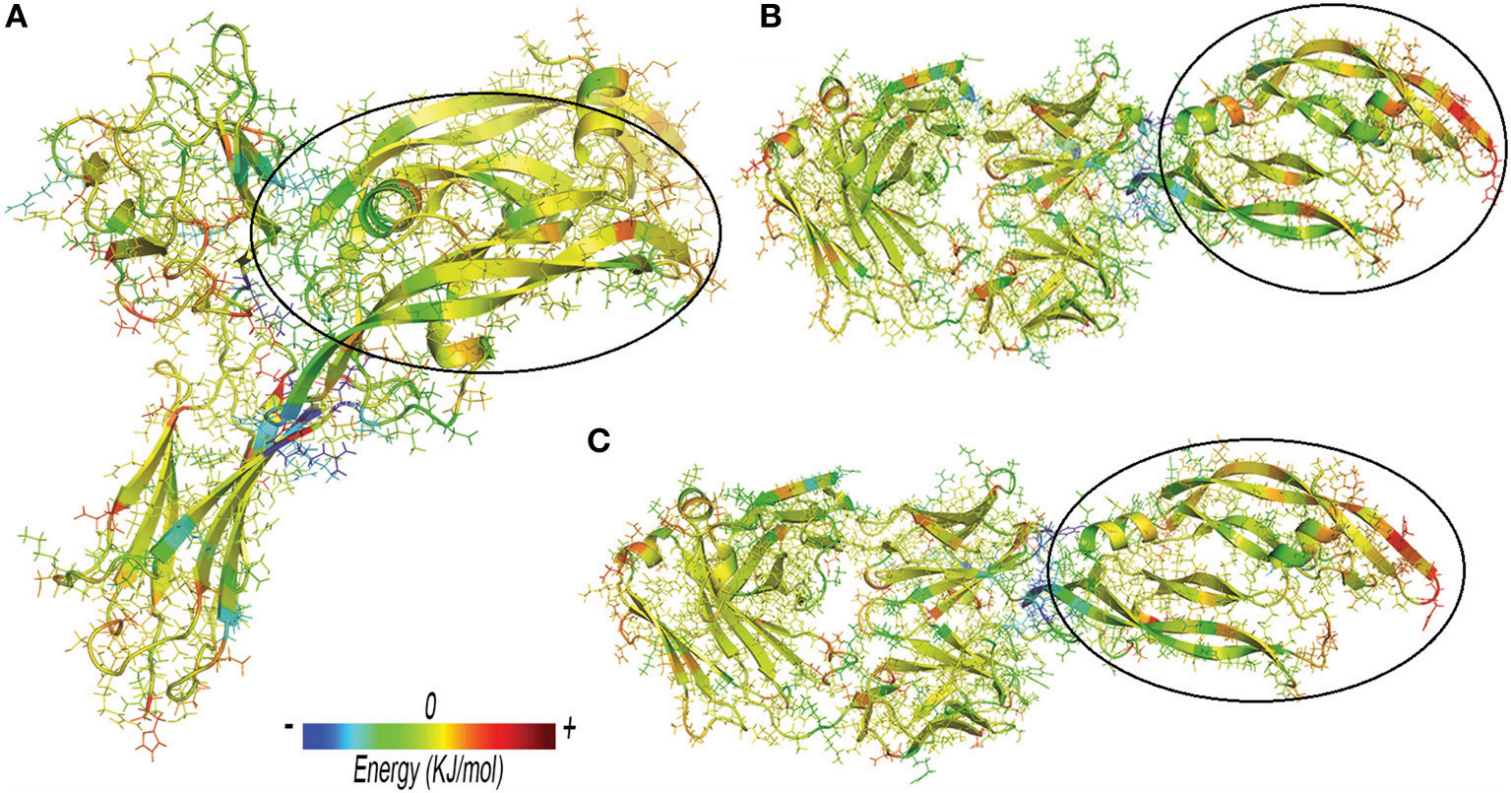
**Three-dimensional projection of energy decomposition results. (A)** VEGFR1d2_R2d3/VEGFA. **(B)** Fab-bevacizumab/VEGFA. **(C)** Ranibizumab/ VEGFA. Stabilizing effect of residues decreases from blue (stabilizing negative energy) to red (destabilizing positive energy). Green or yellow identify neutral (close to zero) contribution to the binding free energy. VEGFA bound to anti-VEGFA agents is highlighted by a black circle. Arrows highlight the areas of proteins with high RMSF.

Anti-VEGF/VEGFA complexes were further analyzed by looking at residues that favorably contribute to ΔE_binding_, by means of energy decomposition calculation. Results were visualized in terms of b-factor (Figure 6). “Hot-spots,” i.e., residues that contribute significantly to complex stabilization (Clackson and Wells, 1995), were identified. As expected, the area that shows the highest stabilization was identified in the contact surface between anti-VEGF and VEGFA. Table 6 shows residues of anti-VEGFs that contribute to stabilization of complexes. The mutations (Ser105Thr; His101Tyr, Asn31His) carried out on Fab-bevacizumab to obtain ranibizumab (Yu et al., 2011), resulted in about a two-fold higher energy stabilization for the ranibizumab/VEGFA complex (Table 6). Interestingly, the stabilizing residues of VEGFR1d2_R2d3 bound to VEGFA are all basic amino acids, confirming the substantial contribute of the electrostatic contribution to the overall ΔE_binding_, as mentioned above.

**Table 6:** **Energy decomposition of predicted ΔE_binding_**.

**anti-VEGF**	**Favorable contributions of residues in anti-VEGF binding domain**
Ranibizumab	Thr 105 (−56), Tyr 101 (−115), His 31(−129)
Fab-bevacizumab	Ser 105 (−44), His 101 (−82), Asn 31 (−22)
VEGFR1d2_R2d3	Arg 154 (−171), Lys 157 (−190), Lys 165(−157), Lys 166 (−129), Arg 128 (−123), Lys 72 (−112), Lys 43 (−118), Lys 89 (−93), Arg 96 (−167)

*Values in brackets correspond to energy contribution of the residues (KJ/mol)*.

## Discussion

The main object of this study was the computational analysis, at molecular level, of binding between VEGFA and the three available anti-VEGF drugs, namely ranibizumab, bevacizumab (Fab-bevacizumab) and aflibercept (VEGFR1d2_R2d3). We have limited our study to interaction between VEGFA and binding domains of the above mentioned anti-VEGF drugs, excluding the Fc fragment of aflibercept and bevacizumab, because the Fc fragment does not seem to influence the pharmacodynamic properties of these drugs (Stewart, 2014b). Wu et al. (2013) already reported the modeling of cobercept (Li et al., 2014), which binds VEGFA with domains VEGFR1d2_R2d3_R2d4, however, no studies have analyzed in detail the energy components contributing to the complex VEGFR1d2_R2d3/VEGFA or compared complexes of different anti-VEGF agents. Therefore, the present study, for the first time, compares the interaction of the three different anti-VEGF agents with VEGFA; this is particularly relevant, considering that aflibercept is structurally unrelated to the other two agents. The entire computational study was carried out with open source tools and software packages.

Protein-protein docking, carried out with PyDock, was the first step. The rough energetic evaluation of complexes predicted by PyDock showed substantial difference between VEGFR1d2_R2d3, ranibizumab and Fab-bevacizumab, VEGFR1d2_R2d3/VEGFA being stabilized mainly by electrostatic energy, whereas the other two complexes were stabilized by VdW and desolvation energy. MD simulation (GROMACS) combined with MM-PBSA calculation (g_mmpbsa tool) was the second step. All MD simulations have been analyzed over a time sufficient to reach relative conformational minimum. Some systems (unbound VEGFR1d2_R2d3 and Fab-bevacizumab/VEGFA) showed high RMSD fluctuations, though secondary structure was conserved and cosine content of eigenvectors was low, indicating correct conformational sampling. MM-PBSA calculations confirmed most of results obtained with PyDock, such as the contribution of electrostatic energy to stability of VEGFR1d2_R2d3/VEGFA and the contribution of Van der Waals interaction energy to ranibizumab/VEGFA and Fab-bevacizumab/VEGFA. Furthermore, MM-PBSA provided energy contributions to ΔE_binding_ in good agreement with experimental binding data (Papadopoulos et al., 2012). MM-PBSA calculation carried out with g_mmpbsa seems at least as successful as other existing tools for analysis of protein-protein docking and MD (Spiliotopoulos et al., 2012; Corrada and Colombo, 2013), but has the advantage of being implemented for GROMACS output files. We obtained a good correlation between experimental K_D_ and apolar desolvation energy (ΔG_apolar_), in accordance to the leading role of solvent exclusion, which is strictly related to the hydrophobic effect of inter- and intra-molecular assembly (Richmond, 1984; Chandler, 2005). Breaking down of ΔE_binding_ helped the identification of several features of complexes. The kinetics of aflibercept/VEGFA binding has been found to be characterized by fast K_on_, which is consistent with substantial favorable electrostatic forces (Papadopoulos et al., 2012). Ranibizumab/VEGF-A had shown low experimental K_off_ (Papadopoulos et al., 2012), i.e., long lasting binding, and this could be related to higher number of contacts and H-bonds and less conformational freedom compared to the other two analyzed complexes (Copeland, 2011). The residue-based RMSF confirmed that upon binding ranibizumab stabilizes VEGFA in comparison to Fab-bevacizumab. VEGFR1d2_R2d3 also stabilizes VEGFA; however, the C-terminal and the N-terminal of VEGFR1d2_R2d3 are characterized by high RMSF, which may account for the higher K_off_ of aflibercept. Furthermore, the g_mmpbsa tool allowed us to identify the residues that favorably contribute to binding energy. A similar approach was carried out by Corrada and Colombo (2013) who have studied correlation of energetic parameters with affinity maturation of 17 variants of bevacizumab bound to VEGFA. A previous study reports two single nucleotide polymorphisms (SNPs) that may influence the binding at VEGFR2 (Wang et al., 2007):

- SNP1192G/A (rs2305948, in exon 7), that corresponds to a mutation Val279Ile in domain 3 of VEGFR2;
- SNP1719A/T (rs1870377, in exon 11), that corresponds to a mutation Q472H in domain 5 of VEGFR2.

SNP1192G/A is more interesting for our study because is located in the domain 3 of VEGFR2, included in aflibercept, and precisely in a beta sheet (one of the two anti-parallel beta sheets) where the residue interacts with other hydrophobic residues. Valine and isoleucine are both hydrophobic, however the isoleucine is more bulky than valine, and Val279Ile mutation might influence the stability of the beta sheets of domain 3. This mutation, along to other SNPs of VEGFRs, is worthy to be studied with the computational approach hereby described.

A recent binding study (Yang et al., 2014) reported an affinity of ranibizumab for VEGFA higher than that of aflibercept. At variance with computational studies, however, experimental binding data appear significantly influenced by the methodology used (Wang and Yang, 2013; Yadav et al., 2014). We have found correspondence between predicted ΔE_binding_ and contributing energy terms to the kinetic and affinity parameters of ranibizumab, bevacizumab and aflibercept, measured by Surface Plasmon Resonance (SPR) by Papadopoulos et al. (2012), who used captured anti-VEGF agents in the matrix and free ligands (VEGFA, VEGFB and PlGF) in the eluent. In contrast, the other SPR setup has used captured VEGFA and free anti-VEGF agents. In this latter setup, the finding that affinity of ranibizumab for VEGFA results greater than that of aflibercept is likely to depend on the blocked access of aflibercept to both ends of VEGFA (Yang et al., 2014).

Protein contact network approach provides a complementary analysis of evolution time for different topological properties of complexes. Fluctuations around a relative conformational minimum were characterized by properties that varied according to given constraints, whereas the energetic descriptor of proteins (dG_solv_) did not change over the time, indicating that MD frames represented relative conformational minimum. The clustering of protein contact network revealed in the VEGFR1d2_R2d3/VEGFA that interaction wiring of VEGFA was conserved in comparison to the other two complexes, where the VEGFA network was altered. Furthermore, clustering of protein contact network of ranibizumab/VEGFA and Fab-bevacizumab/VEGFA was similar but not identical, because VEGFA showed a greater number of long-range interactions toward ranibizumab, in comparison to Fab-bevacizumab. Protein contact networks are built mainly considering VdW interactions; the observation that clustering of VEGFA in ranibizumab/VEGFA and Fab-bevacizumab/VEGFA was altered may be accounted for the higher contribution of VdW in such complexes, as observed with docking and MM-PBSA.

Some controversy exists about the correct length of MD (Dror et al., 2010; Genheden and Ryde, 2010, 2012). Long time scale simulation, in the micro- to millisecond range, is necessary whenever the phenomena observed are in evolution (e.g., binding and unbinding processes, protein folding, conformation transition) (Dror et al., 2010). In the present study we have simulated preformed macromolecular complexes in a water environment in order to carry out MM-PBSA, i.e., analysis of binding energy. To this end, the nanosecond scale seems adequate. Within this time scale, it has been shown that MM-PBSA carried on short (20 ns) independent replicas gives results comparable to a single longer simulation (Genheden and Ryde, 2010, 2012). Furthermore, longer simulations certainly increase sampling size of MD, but would not affect MM-PB(GB)SA results (Genheden and Ryde, 2012).

Our data may provide a theoretical basis for understanding differences in affinity for VEGFA of clinically used anti-VEGFs. Such differences may impact the pharmacodynamics and the therapeutic effectiveness of these biological drugs. However, the clinical outcomes of the *VEGF Trap-Eye: Investigation of Efficacy and Safety in Wet* AMD (VIEW) studies (Schmidt-Erfurth et al., 2014) indicate that ranibizumab and aflibercept are comparable with respect to the number of treatments and visual acuity gains when dosed in identical regimens and concentrations (0.5 mg). The clinical outcomes of *Comparison of Age-related Macular Degeneration Treatments Trials* (CATT) studies (Martin et al., 2012) indicate that ranibizumab and bevacizumab are equally effective on visual acuity over a 2-year period when used in same regimens but at different doses (0.5 and 1.25 mg for ranibizumab and bevacizumab, respectively). The same studies indicate that the proportion of patients with 1 or more systemic serious adverse events was higher with bevacizumab than ranibizumab (39.9 vs. 31.7%; adjusted risk ratio, 1.30; 95% CI, 1.07–1.57; *P* = 0.009). In general, affinity alone, even when it is assessed with high accuracy, does not always straightly correlate with clinical outcome. In fact, between affinity and clinical outcomes there are potency, dose, and regimen parameters to take into account in order to translate *in silico, in vitro*, and *in vivo* data from bench to bedside.

Here we presented *in silico* data of anti-VEGF/VEGFA complexes showing that they considerably differ both in terms of molecular interactions and stabilizing energy. Detailed understanding of such drug-target interactions may help in developing novel biological drugs.

### Conflict of interest statement

The authors declare that the research was conducted in the absence of any commercial or financial relationships that could be construed as a potential conflict of interest.

## Abbreviations

VEGF(A,B,C…): Vascular endothelial growth factor and its isoforms
VEGFR (1, 2…): VEGF receptor and its isoforms
anti-VEGF: Drugs that block VEGF
AMD: Age related macular degeneration
DME: Diabetic macular edema
VEGFR1d2_D2d3: Aflibercept's binding domain
Fab-bevacizumab: Bevacizumab's binding domain
Fab: Fragment antigen-binding
Fc: Fragment crystallizable region
Ig: Human immune globulin
MD: Molecular dynamics
g_mmpbsa: Tool for molecular mechanics Poisson Boltzmann Surface Area calculations (MM-PBSA).
