# Erratum: Molecular features of interaction between VEGFA and anti-angiogenic drugs used in retinal diseases: a computational approach

**DOI:** 10.3389/fphar.2016.00165

**Published:** 2016-06-06

**Authors:** Frontiers Production Office

**Affiliations:** Frontiers Production Office, FrontiersLausanne, Switzerland

**Keywords:** ranibizumab, bevacizumab, aflibercept, diabetic retinopathy, molecular dynamics

Reason for Erratum:

Due to a typesetting error, the article was published with incorrect values in Table 4. The publisher apologizes for this error and the correct version of Table 4 appears below. This error does not change the scientific conclusions of the article in any way.

**Table 4:** **MM-PBSA results compared to experimental binding parameters**.

**Complex**	**Binding parameters**			**MM-PBSA energy terms (KJ/mol)**				
	***K*_on_/10^5^ (M^−1^ s^−1^)**	***K*_off_/10^−5^ (s^−1^)**	***K*_D_ (pM)**	**ΔE_binding_**	**ΔE_VdW_**	**ΔE_electrostatic_**	**ΔG_Polar_**	**ΔG_Apolar_**
Ranibizumab/VEGFA	1.60	0.73	46	−760 ± 40	−418 ± 5	−160 ± 20	410 ± 30	−592 ± 7
Fab-bevacizumab/VEGFA	5.30	3.10	58	−807 ± 30	−362 ± 10	−252 ± 20	343 ± 30	−536 ± 7
VEGFR1d2_R2d3/VEGFA	410	2.01	0.49	−1440 ± 90	−307 ± 50	−1433 ± 100	1050 ± 100	−750 ± 40

Kinetic and binding parameters are from Papadopoulos et al. (2012).
