# Supplementary material for: Molecular features of interaction between VEGFA and anti-angiogenic drugs used in retinal diseases: a computational approach
Source: Front Pharmacol. 2015 Oct 29;6:248. doi: 10.3389/fphar.2015.00248 (PMC4624855; doi:10.3389/fphar.2015.00248)
Supplement: Supplementary file 9 [file Presentation1.PDF]

Supplemental Material

Molecular features of interaction between VEGFA and anti-angiogenic drugs used in retinal diseases: a computational approach

Chiara Bianca Maria Platania[1], Luisa Di Paola[2], Gian Marco Leggio [1], Giovanni Luca Romano[1], Filippo Drago [1], Salvatore Salomone[1], Claudio Bucolo [1]*

* correspondence: Claudio Bucolo, claudio.bucolo@unict.it

[1]Department of Biomedical and Biotechnological Sciences, Section of Pharmacology and Biochemistry, School of Medicine, University of Catania, Catania, Italy; [2] School of Engineering, University CAMPUS BioMedico, Roma, Italy.

1.Supplementary Data

1.1. Preliminary molecular dynamics of aflibercept binding domain.

A preliminary molecular dynamics (MD) of VEGFR1d2_R2d3 was carried out in order to overcome the structural restraints related to building of the model from the template x-ray structure (PDB: 2X1W) and to account for structural flexibility of the hinge connecting R1d2 and R2d3 domains. The binding domain of aflibercept reached a relative conformational minimum within 10 ns of simulation (Fig. S1 B), with an average root mean square fluctuation (RMSD) of about 0.8 nm respect to the initial structure. The radius of gyration of VEGFR1d2_R2d3 decreased during the simulation (Fig. S1 C) and reached a minimum at 5 ns, with a trend similar to the RMSD profile. Frames from 5 to 10 ns were considered equivalent because they belonged to the same conformational minimum. Two frames, whose RMSD was 0.06 nm from comparison, were randomly chosen and used for protein-protein docking. Fluctuations in RMSD and Rg profiles, in the region between 5-10 ns, are within 0.5-2 Å; these fluctuation are commonly below the X-ray resolution for this reason we consider frames between 5-10 ns as structurally equivalent. Indeed analysis of secondary and cosine content (data not shown for this simulation) confirmed that the conformational sampling is satisfactory for the system.

1.2. Protein-protein docking of VEGFR1d2_R2d3 with VEGFA.

Scores of predicted complexes relative to two randomly chosen MD frames of VEGFR1d2_R2d3 docked to VEGFA were not different, because of low RMSD from their comparison, 0.06 nm. This result confirm the equivalence of the two frames belonging to the same conformational minimum. The complex VEGFR1d2_R2d3/VEGFA was compared either to human native VEGFR2d2_d3 bound to VEGFC (PDB: 2X1W, RMSD 0.3 nm) or VEGFR2d2_d3 bound to VEGFA (PDB: 3V2A, RMSD 0.2 nm). The prediction of the complex VEGFR1d2_R2d3/VEGFA, without MD optimization of VEGFR1d2_R2d3, gave a less favorable score (Table 1 in the manuscript). We also obtained the VEGFR1d2_R2d3/VEGFA 2:1 predicted complex; as shown in Fig. S2 B; the main deviation from the x-ray structure PDB: 2X1W involved an outward bending of the domain 3 from the core of the complex.

1.3. Secondary structure of VEGFR1d2_R2d3 and Fab-bevacizumab/VEGFA.

Secondary structure evolution of protein during MD has been analyzed with the timeline tool of Visual Molecular Dynamics software VMD 1.9 (Humphrey et al., 1996). The secondary structure of VEGFR1d2_R2d3 is substantially conserved during the simulation, especially in correspondence of extended β strands. VEGFR1d2_R2d3 showed structural flexibility in correspondence of loops in the R1d2 domain. These loops inter-converted from turn to coil conformation during the simulations. R2d3 domain showed low structural flexibility. Conversions of one loop from turn to 3-helix were also found. Several β-bridges were found in the R1d2 domain; the main function of β-

bridges has been accounted for conformational adaptations upon binding (Guan et al., 2004). The high flexibility of R1d2 domain, might have a role in stability of VEGFR1d2_R2d3/VEGFA. This structural flexibility would influence positively the association rate, favoring conformation adaptation upon binding; while flexibility would influence negatively dissociation rate increasing K_{off}, due to conformational instability and short residence time (Copeland, 2011).
The secondary structure of Fab-bevacizumab/VEGFA complex was substantially conserved during the simulation; a loop of Fab-bevacizumab ^{102}YYGSSHWYF^{110}, that is in direct contact with VEGFA, showed structure flexibility converting from turn to coil. The same loop in ranibizumab/VEGFA complex conserved turn structure in all simulations, though during simulation temporary conversion to coil is observed similarly to Fab-bevacizumab/VEGFA. ^{102}Y is highlighted for clarity in Fig. S6-7-8.

1.4. PC1 projection into MD trajectory.

Videos have been created loading each PC1 projection to the structure of simulated system using Visual Molecular Dynamics VMD 1.9, on a Desktop PC (12 core Intel i7, 64 GB RAM, two GeForce GTX 680-SLI).

- PC1_fab_bevacizumab.mpg: PC1 projected on trajectory of fab-bevacizumab
- PC1_fab_bevacizumab_vegfa.mpg: PC1 projected on trajectory of fab-bevacizumab/VEGFA complex
- PC1_ranibizumab.mpg: PC1 projected on trajectory of ranibizumab
- PC1_ranibizumab_vegfa.mpg: PC1 projected on trajectory of ranibizumab/VEGFA complex
- PC1_vegfaSIDE.mpg: PC1 projected on trajectory of VEGFA, side view.
- PC1_vegfaTOP.mpg: PC1 projected on trajectory of VEGFA, top view.
- PC1_VEGFR1d2_R2d3.mpg: PC1 projected on trajectory of VEGFR1d2_R2d3.
- PC1_VEGFR1d2_R2d3_vegfa.mpg: PC1 projected on trajectory of VEGFR1d2_R2d3/VEGFA complex.

1.5. Protein contact networks of unbound systems.

The Analysis of MD trajectories with protein contact network approach has been carried out. The unbound systems showed time-invariance of all key descriptors (Table S1). As for complexes anti-VEGF/VEGFA, the graph energy *E* correlates strongly and positively with adeg; this result confirm that E would indicate interaction potential of network.
Clustering of contact networks of unbound systems has been carried out with a spectral clustering algorithm (Fig S9). Clustering partitioning of VEGFA (Fig S9 A) shows two intermingled clusters of residues (nodes). In VEGFR1d2_R2d3 clusters corresponds to domain N-terminal R1d2 and C-terminal R2d3 domains of aflibercept binding domain (VEGFR1d2_R2d3) (Fig S9 B). Two intermingled clusters have been identified in Fab-bevacizumab and ranibizumab; these two anti-VEGFs shows similar clustering of protein contact networks.

2. Supplementary tables and figures.

2.1.

Table S1. Correlation analysis of topological parameters of unbound systems.

	**Ranibizumab**					**Fab-bevacizumab**				
	**t**	**adeg**	**asp**	**E**	**dG_{solv}**	**t**	**adeg**	**asp**	**E**	**dG_{solv}**
**t**	-	-0.40	0.21	-0.45	0.24	-	0.22	0.05	0.10	-0.08
**adeg**	-	-	-0.34	*0.95*	-0.51	-	-	-0.19	*0.89*	-0.23
**asp**	-	-	-	-0.33	0.31	-	-	-	-0.31	0.11
**E**	-	-	-	-	*-0.52*	-	-	-	-	-0.20
**dG_{solv}**	-	-	-	-	-	-	-	-	-	-
	**VEGFR1d2_R2d3**					**VEGFA**				
	**t**	**adeg**	**asp**	**E**	**dG_{solv}**	**t**	**adeg**	**asp**	**E**	**dG_{solv}**
**t**	-	0.24	*0.64*	0.12	0	-	-0.35	0.14	-0.36	0.08
**adeg**	-	-	-0.11	*0.91*	-0.09	-	-	-0.54	*0.92*	-0.22
**asp**	-	-	-	-0.20	0.28	-	-	-	-0.55	0.16
**E**	-	-	-	-	-0.10	-	-	-	-	-0.22
**dG_{solv}**	-	-	-	-	-	-	-	-	-	-

2.2. Supplementary Figures

Figure Legends

Fig. S1. Modeling and preliminary molecular dynamics of VEGFR1d2_R2d3. A. Superimposition of aflibercept's binding domain (green) over the x-ray structure of VEGFR2d2_d3/VEGFC complex (grey). B. root-mean-square deviation (RMSD) of unbound VEGFR1d2_R2d3; C. radius of gyration of VEGFR1d2_R2d3 during the simulation.

Fig. S2. Superimposition of predicted VEGFR1d2_R2d3/VEGFA with human native VEGFR2d2_d3/VEGFC complex. A. 1:1 predicted complex; B. 2:1 predicted complex. VEGFR2d2_d3/VEGFC complex (gray); VEGFR1d2 domain (green); VEGFR2_d3 domain (dark blue); VEGFA (orange); VEGFC (cyan).

Fig.S3. Secondary structure evolution within 40 ns of simulation of replica 1 of VEGFR1d2_R2d3. Notations for secondary structure elements are: H = α-helix, B = residue in isolated β-bridge, E = extended strand, participates in β ladder, G = 3-helix (310 helix), I = 5 helix (π-helix), T = hydrogen bonded turn, S = bend, C=coil.

Fig. S4. Secondary structure evolution within 40 ns of simulation of replica 2 of VEGFR1d2_R2d3. Notations for secondary structure elements are: H = α-helix, B = residue in isolated β-bridge, E = extended strand, participates in β ladder, G = 3-helix (310 helix), I = 5 helix (π-helix), T = hydrogen bonded turn, S = bend, C=coil.

Fig S5. Secondary structure evolution within 40 ns of simulation of replica 3 of VEGFR1d2_R2d3. Notations for secondary structure elements are: H = α-helix, B = residue in isolated β-bridge, E = extended strand, participates in β ladder, G = 3-helix (310 helix), I = 5 helix (π-helix), T = hydrogen bonded turn, S = bend, C=coil.

Fig. S6. Secondary structure evolution within 40 ns of simulation of replica 1 of Fab-bevacizumab/VEGFA. Notations for secondary structure elements are: H = α-helix, B = residue in

isolated β-bridge, E = extended strand, participates in β ladder, G = 3-helix (310 helix), I = 5 helix (π-helix), T = hydrogen bonded turn, S = bend, C=coil.

Fig. S7. Secondary structure evolution within 40 ns of simulation of replica 2 of Fab-bevacizumab/VEGFA. Notations for secondary structure elements are: H = α-helix, B = residue in isolated β-bridge, E = extended strand, participates in β ladder, G = 3-helix (310 helix), I = 5 helix (π-helix), T = hydrogen bonded turn, S = bend, C=coil.

Fig. S8. Secondary structure evolution within 40 ns of simulation of replica 3 of Fab-bevacizumab/VEGFA. Notations for secondary structure elements are: H = α-helix, B = residue in isolated β-bridge, E = extended strand, participates in β ladder, G = 3-helix (310 helix), I = 5 helix (π-helix), T = hydrogen bonded turn, S = bend, C=coil.

Fig. S9 Clustering of protein contact networks. Distribution of clusters along sequence in a matricial space. Partition color maps of A. VEGFA, B. VEGFR1d2_R2d3, C. Fab-bevacizumab, D. ranibizumab. Residues (nodes) belonging to the same cluster have the same color, long projections "whiskers" in the map represent residues shifting to a different cluster with respect to that of neighbors in sequence. The background is deep blue color and characterizes residues, which are not the same cluster.

References

Copeland, R.A. (2011). Conformational adaptation in drug-target interactions and residence time. *Future medicinal chemistry* 3, 1491-1501.

Guan, C., Kumar, S., Kucera, R., and Ewel, A. (2004). Changing the enzymatic activity of T7 endonuclease by mutations at the beta-bridge site: alteration of substrate specificity profile and metal ion requirements by mutation distant from the catalytic domain. *Biochemistry* 43, 4313-4322.

Humphrey, W., Dalke, A., and Schulten, K. (1996). VMD: Visual molecular dynamics. *Journal of Molecular Graphics & Modelling* 14, 33-38.
