# Supplementary figures and images for: Molecular features of interaction between VEGFA and anti-angiogenic drugs used in retinal diseases: a computational approach
Source: Front Pharmacol. 2015 Oct 29;6:248. doi: 10.3389/fphar.2015.00248 (PMC4624855; doi:10.3389/fphar.2015.00248)

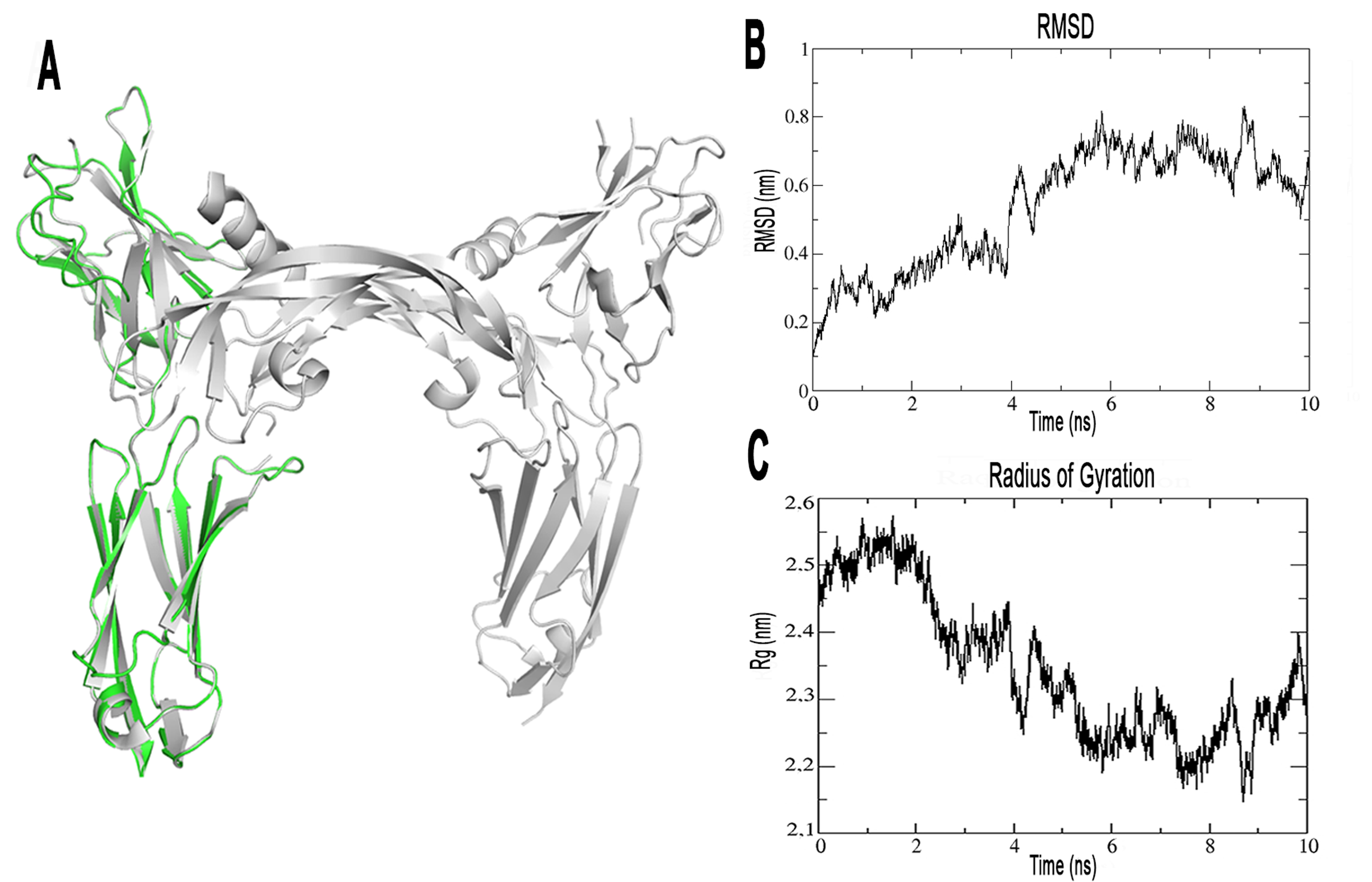

Supplement: Supplementary file 10 [file Image1.TIF]

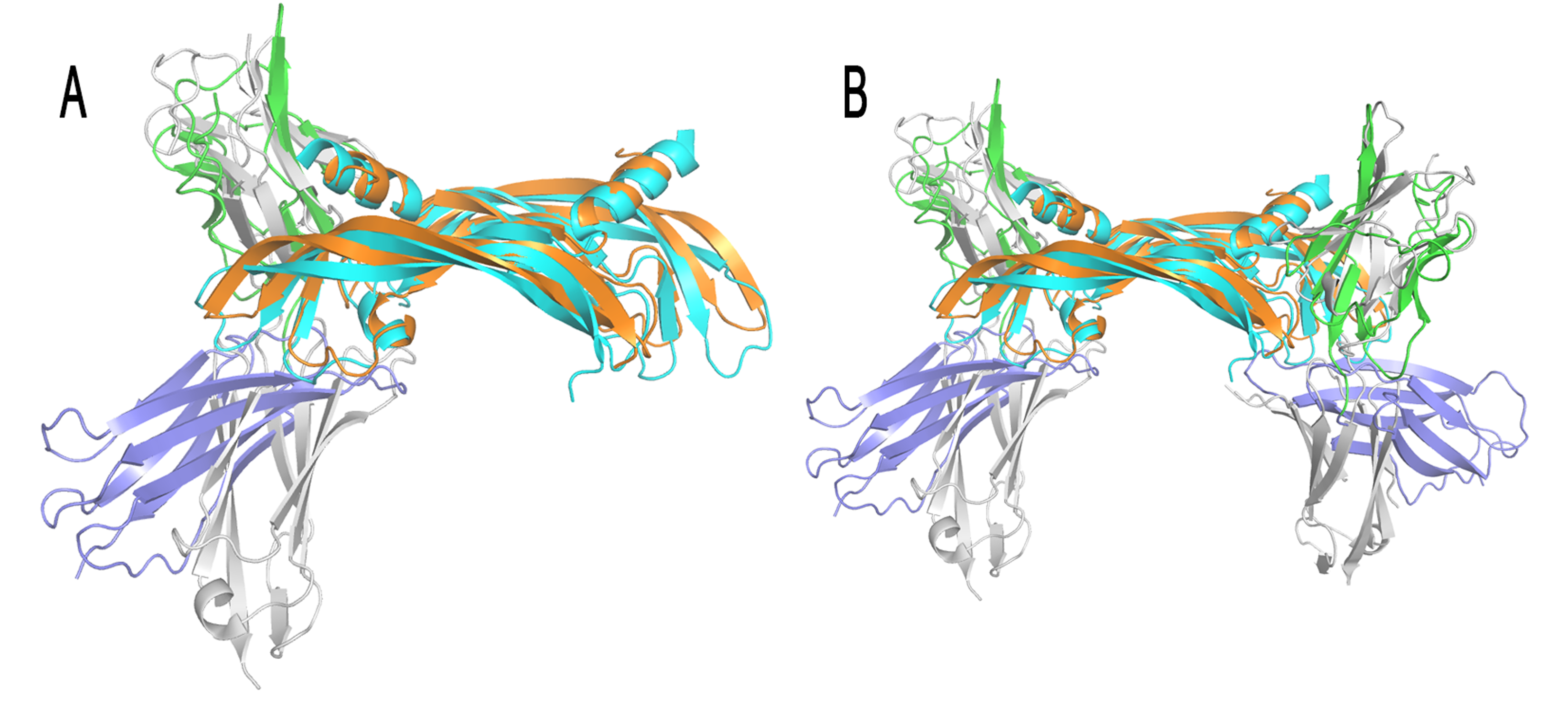

Supplement: Supplementary file 11 [file Image2.TIF]

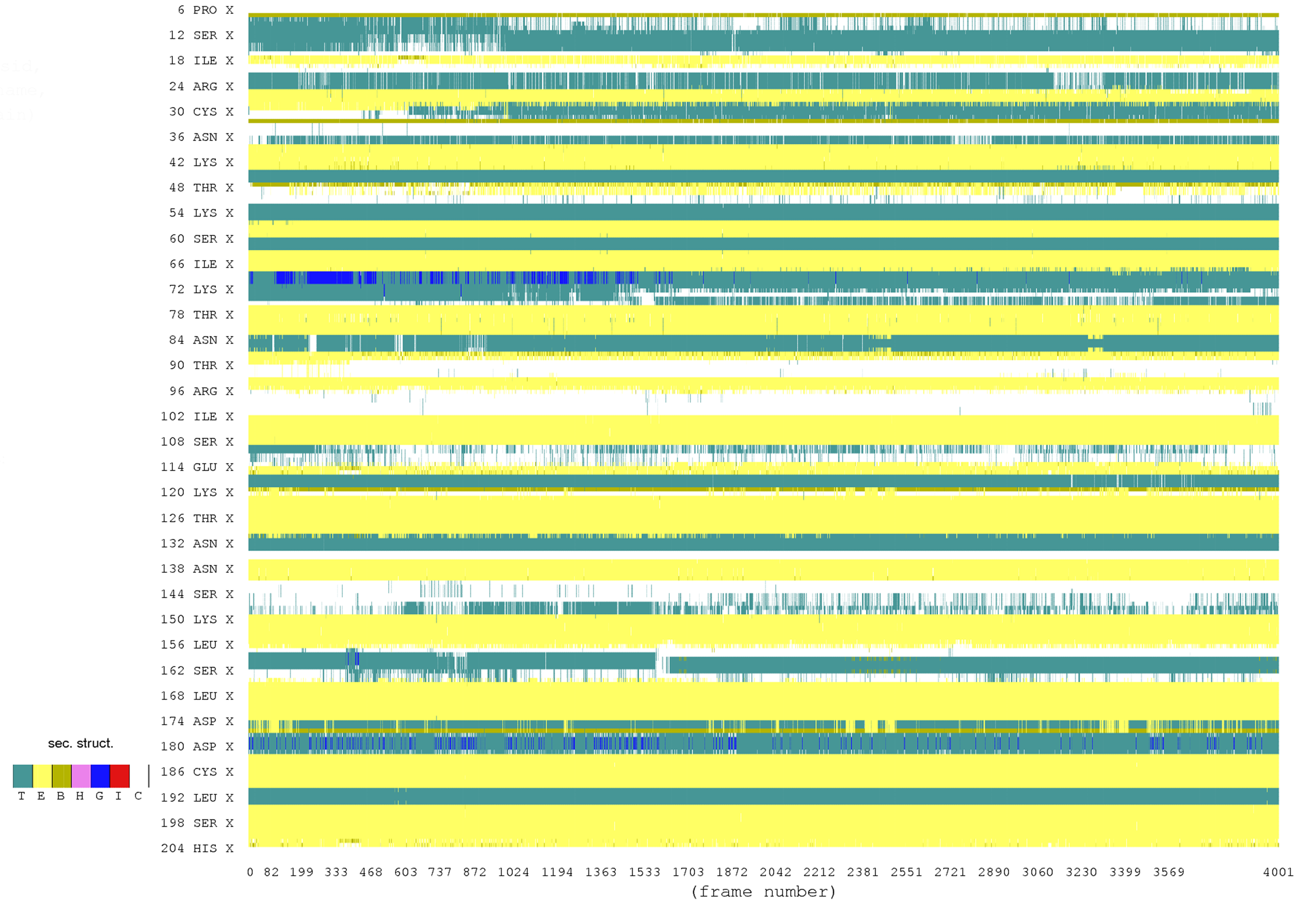

Supplement: Supplementary file 12 [file Image3.TIF]

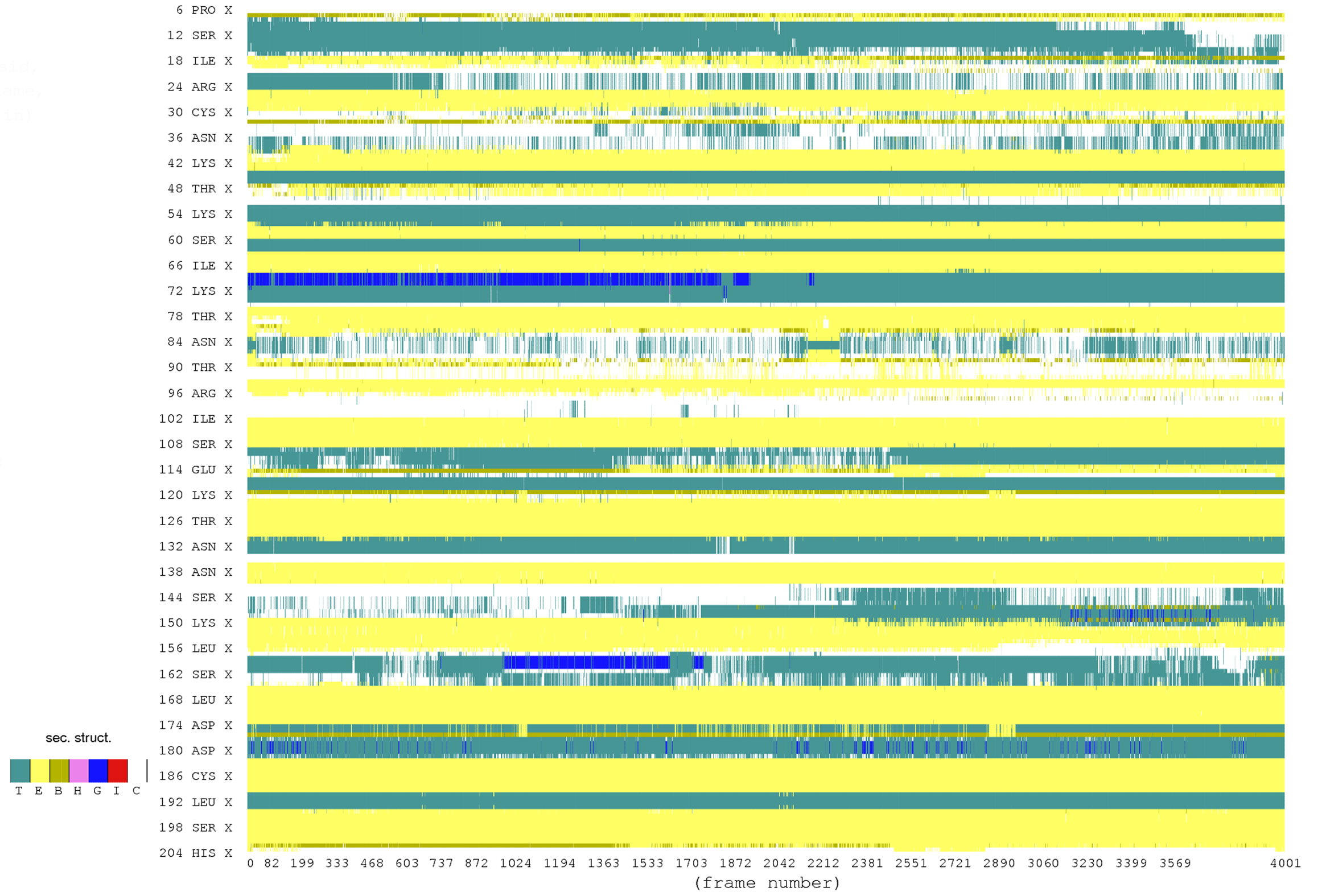

Supplement: Supplementary file 13 [file Image4.TIF]

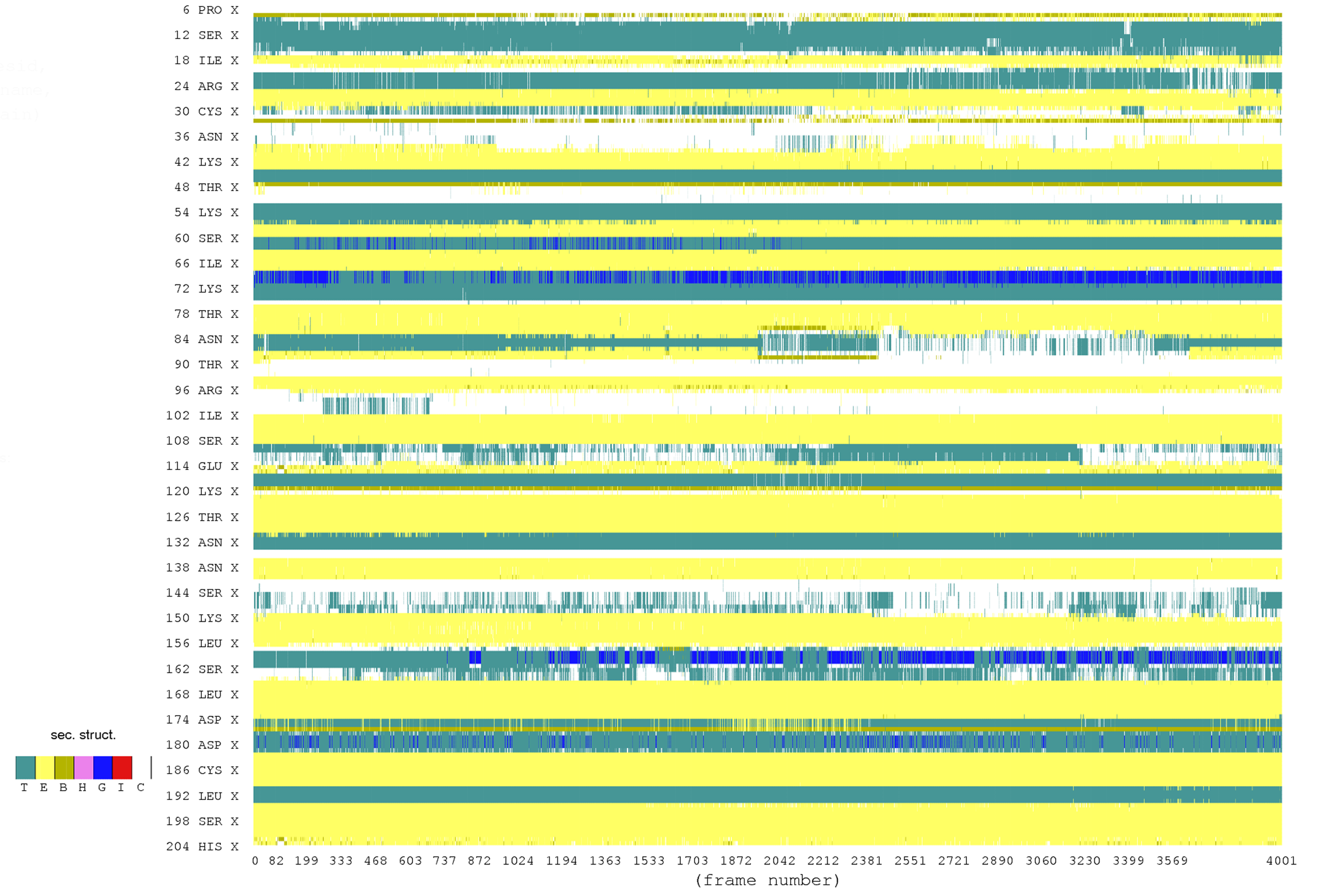

Supplement: Supplementary file 14 [file Image5.TIF]

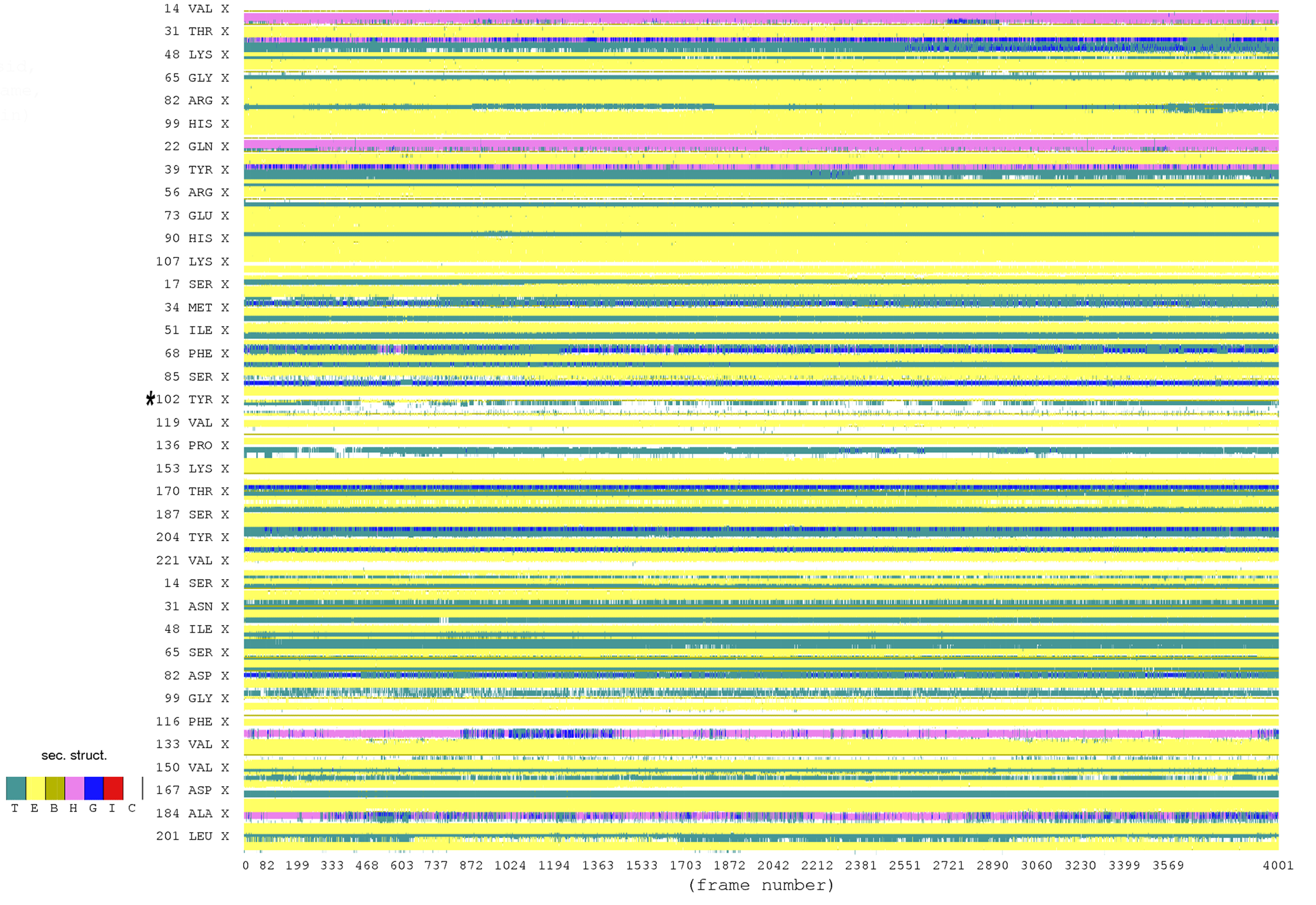

Supplement: Supplementary file 15 [file Image6.TIF]

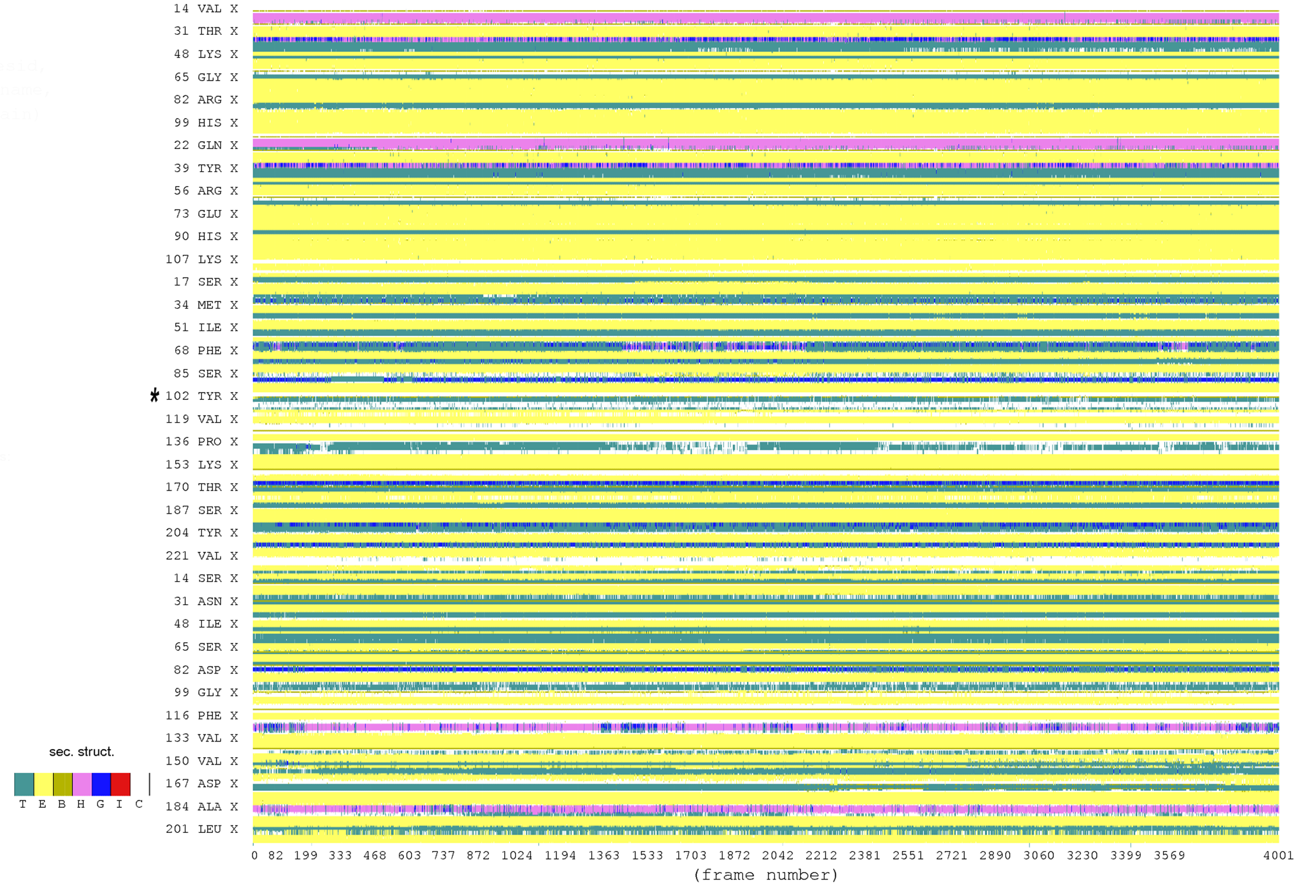

Supplement: Supplementary file 16 [file Image7.TIF]

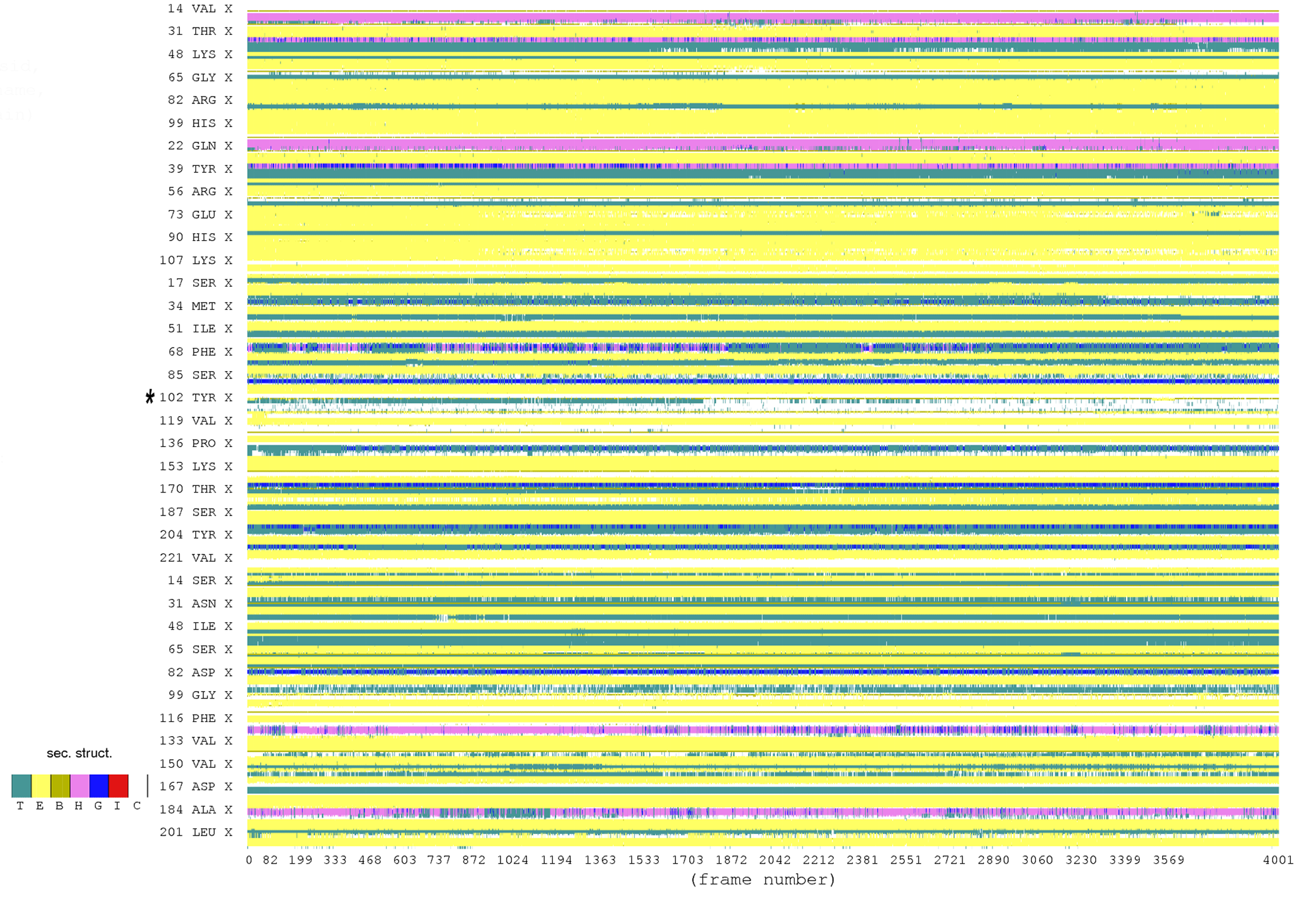

Supplement: Supplementary file 17 [file Image8.TIF]

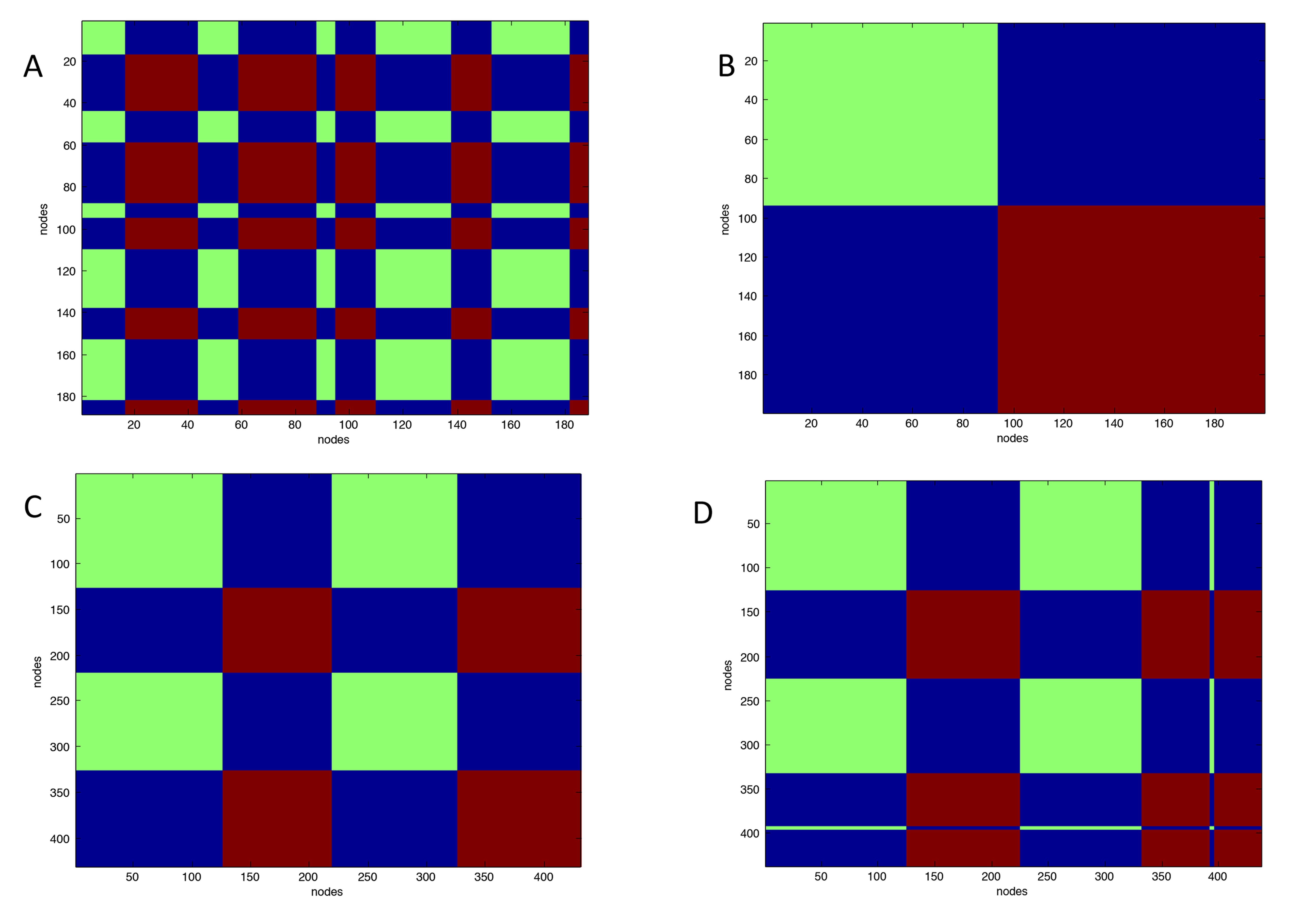

Supplement: Supplementary file 18 [file Image9.TIF]
